# Transepidermal Water Loss in Oral Food Challenges in Children With Peanut Allergy: A Randomized Clinical Trial

**DOI:** 10.1001/jamanetworkopen.2025.43371

**Published:** 2025-11-14

**Authors:** George E. Freigeh, Kelly M. O’Shea, Jonathan P. Troost, Bridgette Kaul, Lea M. Franco, Charles F. Schuler

**Affiliations:** 1 Division of Allergy and Clinical Immunology, Department of Internal Medicine, University of Michigan, Ann Arbor; 2 Division of Pediatric Hematology and Oncology, Department of Pediatrics, University of Michigan, Ann Arbor; 3 Mary H. Weiser Food Allergy Center, Ann Arbor, Michigan; 4 Michigan Institute for Clinical and Health Research, University of Michigan, Ann Arbor

## Abstract

**Importance:**

Food allergy is a prevalent chronic medical condition in children, with limited diagnostic options. Transepidermal water loss (TEWL) is a measure of skin barrier function with emerging utility in atopy and food allergy.

**Objective:**

To determine whether use of TEWL as a stopping criterion can prevent anaphylaxis during a peanut oral food challenge (OFC).

**Design, Setting, and Participants:**

This was a single-center randomized clinical trial of children aged 6 months to 5 years with a history of a clinical peanut allergic reaction and positive peanut skin prick and/or serum immunoglobulin E testing. All participants underwent peanut OFC at an allergy referral clinic. The study began May 1, 2023, and ended August 31, 2024.

**Intervention:**

The intervention was stopping OFC based on a 1 g/m^2^/h rise in TEWL plus 1 objective allergic symptom observed by the physician during OFC. Standard OFC stopping criteria or the intervention criteria, whichever came first, were used in the intervention group. OFC stopping criteria from standard research were used in the control group. This was a single-intervention study.

**Main Outcome and Measures:**

Rate of anaphylaxis, defined as a Consortium for Food Allergy Research (CoFAR) score of 2 or higher, compared between the intervention and control groups using 4 anaphylaxis criteria approaches: CoFAR, Brighton Collaboration, Food Allergy and Anaphylaxis Network (FAAN), and World Allergy Organization (WAO).

**Results:**

A total of 40 participants were included in the final analysis. The mean (SD) age of participants was 31.8 (16.18) months, with a median of 32.5 (range, 9-62) months; 23 (58%) were male. Using CoFAR criteria, among reactors, the anaphylaxis rate was 10 of 16 (63%; 95% CI, 39%-86%) in the intervention group compared with 14 of 14 (100%; 95% CI, 100%-100%) in the control group (*P* = .02). Using WAO criteria, 2 of 16 participants were reactors in the intervention group (anaphylaxis rate, 13%; 95% CI, 0%-29%) compared with 8 of 14 (57%; 95% CI, 31%-83%) in the control group (*P* = .02). There was no significant difference when comparing anaphylaxis rates using Brighton or FAAN criteria (eg, rate of 56% [95% CI, 32%-81%] vs 71% [95% CI, 48%-95%] in intervention vs control participants using Brighton criteria [*P* = .47]). The median CoFAR score in reactors in the intervention vs control groups was 1.8 (IQR, 1.0-2.0) vs 2.6 (IQR, 2.0-3.0) (*P* = .006). Eight of 16 participants (50%) in the intervention vs 12 of 14 (86%) in the control group required epinephrine (*P* = .06).

**Conclusions and Relevance:**

In this randomized clinical trial, prospective use of TEWL as a stopping criterion reduced anaphylaxis rates during OFC in children with peanut allergy, suggesting use of TEWL could make OFC safer and more accessible. Replicating this study in a larger, multicenter population is a reasonable next step.

**Trial Registration:**

ClinicalTrials.gov Identifier: NCT05696236

## Key Points

**Question:**

Can transepidermal water loss (TEWL) as a measure of skin barrier function be used to reduce anaphylaxis rates and severity during oral food challenges in children with peanut allergy?

**Findings:**

In this randomized clinical trial that included 40 children aged 6 months to 5 years with peanut allergy, the anaphylaxis rate among participants with an allergic reaction during a peanut oral food challenge was lower in the group for which TEWL was used as a stopping criterion (63%) than in the control group (100%).

**Meaning:**

The findings suggest TEWL can augment anaphylaxis prediction during oral food challenges.

## Teaser

This randomized clinical trial investigates whether transepidermal water loss as a measure of skin barrier function can be used as a stopping criterion to reduce anaphylaxis rates during oral food challenges in children with peanut allergy.

## Introduction

Food allergy (FA) remains a common worldwide health issue, affecting approximately 8% of children.^1,2^ Food anaphylaxis accounts for approximately 1% of emergency department visits in the US.^3^ Despite the high disease burden, diagnostic modalities are limited, with high rates of false-positive food-specific skin prick and serum immunoglobulin E (IgE) testing.^4^ The oral food challenge (OFC) remains the gold standard in diagnosing FA.^5^ However, the perceived and actual risks of OFCs limit more widespread use.^6^ Developing novel diagnostic methods and identifying reliable anaphylaxis predictors is a top need in FA.^7^

Transepidermal water loss (TEWL) measures insensible water loss through the skin.^8^ TEWL is measured as water efflux in grams per square meter per hour, and typical values range from 4 to 10 g/m^2^/h.^8^ Any condition that disrupts the skin barrier can increase TEWL, and the measurement is considered a proxy for skin barrier integrity.^8^ There is robust literature examining TEWL in atopic dermatitis (AD), with higher TEWL values in individuals with AD, both in lesional and nonlesional skin.^9,10,11^ It is evident that epithelial function is involved in the development of atopic conditions beyond AD. For example, there is an association of variants in the epithelial protein SPINK5 with FA.^12^

Works including the dual-allergen exposure hypothesis^13^ support examining how the epithelial barrier may change during food anaphylaxis. Our group published an observational study examining real-time TEWL changes during OFCs and found that during reactions, TEWL reliably rose by 1 g/m^2^/h and preceded objective signs of reactions, suggesting a predictive capacity of TEWL for anaphylaxis.^14,15^ This is intriguing given the risk of anaphylaxis associated with OFCs, particularly in the context of expected reactions, such as for clinical trials of FA therapies.^16,17^ Early identification and treatment of anaphylaxis may attenuate reaction severity and adverse outcomes^18,19^; thus, there is a longstanding interest in predicting or detecting anaphylaxis during OFCs. Additionally, any tool that improves OFC safety may ameliorate perceived concerns from practitioners and patients, thereby extending the potential reach and use of OFCs.

Herein we present a pilot study assessing the capacity of TEWL as a prospective, real-time predictor of anaphylaxis. The goal of this study was to determine if real-time monitoring of TEWL could lead to earlier detection of anaphylaxis, leading to less severe reactions.

## Methods

### Study Design and Participant Population

This randomized clinical trial followed the Consolidated Standards of Reporting Trials (CONSORT) reporting guideline. The study protocol is in Supplement 1. This study was approved by the University of Michigan institutional review board. Written consent was obtained by trained research coordinators (B.K., L.M.F.) after appropriate discussion of the study.

The trial began May 1, 2023, and ended August 31, 2024. The study population included participants aged 6 months to 5 years at OFC. Participants had a history of peanut reaction confirmed by an allergist. Testing for peanut sensitization must have been completed within the previous 12 months and have met the 80% likelihood positive predictive value threshold for peanut allergy based on either the skin or blood IgE tests per current literature; for peanut allergy, this requires at least a 3-mm wheal on skin prick testing, total peanut IgE level of 5.0 kUa/L, and Ara h1 or h2 of more than 0.35 kUa/L.^4,20^ Exclusion criteria are detailed in the eMethods in Supplement 2.

Eligible participants were randomized into a control and an intervention group in a 1:1 fashion using permuted block randomization. We varied the block size from 2 to 4 to enhance blinding. Randomization was performed at first visit (initial screening visit) or no later than the day of the OFC. A continuous TEWL monitor (detailed subsequently) was applied to all participants prior to and throughout the OFC.

All participants underwent peanut OFC. The control group used challenge-stopping criteria determined by dose-limiting symptoms per the Consortium for Food Allergy Research (CoFAR).^21^ The intervention group used challenge-stopping criteria based on rise in TEWL of 1 g/m^2^/h plus the presence of 1 objective (ie, directly observable by the adjudicating physician) allergic symptom as included in CoFAR and Brighton criteria^20^ (which alone might not be sufficient to end an OFC) or the presence of dose-limiting symptoms per CoFAR criteria, whichever came first. The TEWL change had to occur in the 2 minutes before the challenge dose vs after the challenge dose was given. This was so that the research coordinator (L.M.F.) monitoring TEWL had a definable range to refer to and because our group’s prior work suggested that the TEWL change occurs at this time.^14^

The participant, the participant’s guardians, the food challenge nurse (B.K.), and the research physicians (G.E.F., K.M.O., C.F.S.) adjudicating the OFC outcome were blinded to the participant’s study group and TEWL measurements. A research coordinator, unblinded to the participant’s study group, monitored the participant’s continuous TEWL measurement from behind a screen. Whenever an objective allergy symptom occurred, the research physician would announce to the unblinded research coordinator that an objective symptom was present. If the participant was in the intervention group and met the TEWL rise criteria, the research coordinator would announce stopping criteria had been met and the OFC would end. No additional doses would be given, and the participant would receive any indicated treatment immediately. Otherwise, the challenge would continue and the unblinded coordinator otherwise remained silent. This process would continue until the patient developed dose-limiting symptoms, met TEWL stopping criteria if in the intervention group, or completed the challenge without reaction. A visual study schematic is in eFigure 1 in Supplement 2.

The primary outcome measurement was a comparison of anaphylaxis rates between groups. We used 4 approaches to make a comparison, including CoFAR,^21^ Brighton Collaboration,^22^ Food Allergy and Anaphylaxis Network (FAAN),^23^ and World Allergy Organization (WAO)^24^ methods. For this study, anaphylaxis was defined as a CoFAR score of 2 or higher out of a score range of 1 to 5. There is no universal definition of anaphylaxis in FA research, though a CoFAR score of 2 or higher indicates involvement of 2 or more organ systems and/or severe symptoms in a single organ (eg, hypotension).^21^ A reaction was defined as fulfilling traditional stopping criteria for a dose-limiting symptom or fulfilling TEWL stopping criteria in the intervention group.

Participant race and ethnicity were ascertained by self-report and included in the analysis because skin tone has been debated as a factor in TEWL results in the past.^11^ Race categories were Asian, Black or African American, White, and other (included undisclosed or not known). Ethnicity categories were Hispanic and non-Hispanic.

### OFC Protocol

OFCs were performed according to published guidelines.^5^ The full OFC protocol is available in the eMethods in Supplement 2.

### TEWL Measurement

All measurements were taken on visually normal skin. Further details on standardization of TEWL measurements are reported elsewhere^14^ and in the eMethods in Supplement 2.

### Statistical Analysis

Continuous variables were described using means with SDs or medians with IQRs and ranges. Categorical variables were described using frequencies and percentages. Comparisons by treatment group were made using Kruskal-Wallis tests for continuous variables and χ^2^ tests for categorical or binary variables. Two-sided α = .05 was used to determine statistical significance. Analyses were performed in SAS, version 9.4 (SAS Institute Inc). A detailed power analysis and worst-case scenario method is in the eMethods in Supplement 2.

## Results

**Figure 1.**
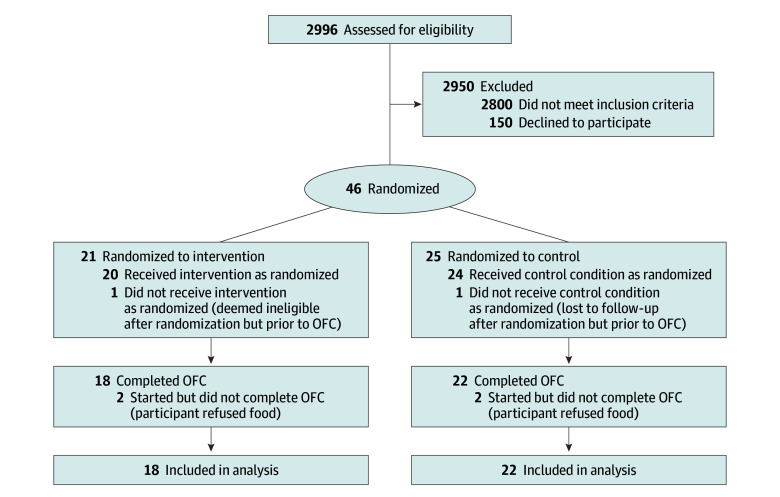
CONSORT Diagram OFC indicates oral feeding challenge.

A total of 46 participants were randomized, 25 to the control group and 21 to the intervention group. In the control group, 22 participants (88%) completed the OFC (1 participant [4%] was deemed ineligible after randomization prior to OFC, and 2 participants [8%] started OFC but did not complete it due to food refusal). In the intervention group, 18 participants (86%) completed the OFC (1 participant [5%] was lost to follow-up prior to OFC and 2 participants [10%] started OFC but did not complete it due to food refusal). Thus, 40 participants were included in the final analysis (Figure 1).

The mean (SD) age of participants was 31.8 (16.2) months and median age was 32.5 months (range, 9-62 months). Seventeen participants (42%) were female and 23 (58%) were male. One participant (2%) was Asian, 1 (2%) was Black or African American, 37 (92%) were White, and 1 (2%) was other race. Two (5%) were Hispanic and 38 (95%) were non-Hispanic ethnicity. The median total peanut serum IgE level and Ara h2 level were 1.3 kUa/L (IQR, 0.6-5.8 kUa/L) and 0.8 kUa/L (IQR, 0.3-5.9 kUa/L), respectively, without significant difference between control and intervention groups. There was a difference in total distribution of peanut skin prick wheal, with the control group having a mean (SD) wheal of 8.8 (3.96) mm and the intervention group having a mean (SD) wheal of 5.1 (2.45) mm (*P* = .003). However, there was no significant difference between groups when comparing the proportions with a peanut wheal 3 mm or larger: 15 of 17 (88%) in the intervention vs 21 of 21 (100%) in the control group (*P* = .11). Demographic information is summarized in the eTable in Supplement 2.

**Figure 2.**
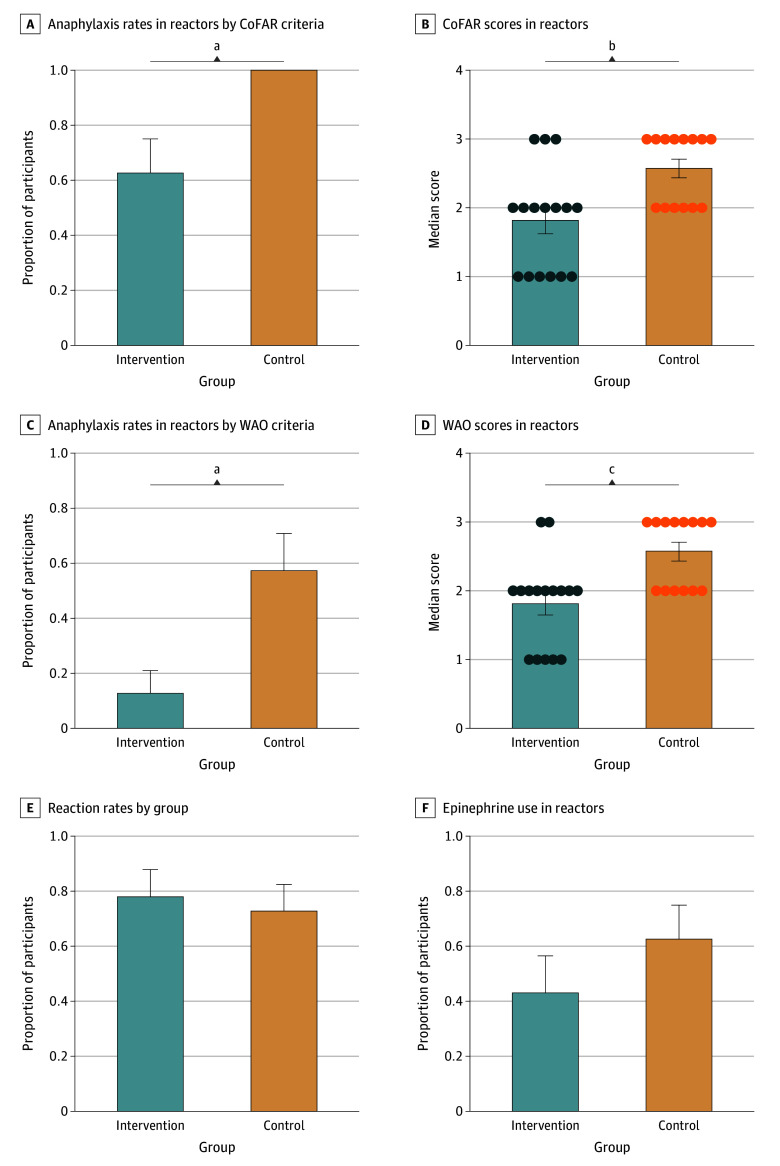
Anaphylaxis Rates, Reaction Rates, and Epinephrine Use in Reactors Statistical analysis used χ^2^ tests and *t* tests. Error bars signify SEMs and circles, individual data points. CoFAR indicates Consortium for Food Allergy Research; WAO, World Allergy Organization. CoFAR score range, 1 to 5, with 2 or higher indicating anaphylaxis. WAO score range, 1 to 5, with higher scores indicating more severe reactions. ^a^*P* = .02. ^b^*P* = .006. ^c^*P* = .002.

The primary outcome was a comparison of anaphylaxis rates. Reaction data are summarized in the Table and Figure 2. Among reactors, the anaphylaxis rate was 10 of 16 (63%; 95% CI, 39%-86%) in the intervention group vs 14 of 14 (100%; 95% CI, 100%-100%) in the control group using CoFAR criteria (*P* = .02) (Figure 2A). Using WAO criteria, 2 of 16 participants were reactors (anaphylaxis rate, 13%; 95% CI, 0%-29%) in the intervention group vs 8 of 14 (anaphylaxis rate, 57%; 95% CI, 31%-83%) in the control group (*P* = .02) (Figure 2C). Using Brighton criteria, 9 of 16 participants were reactors (anaphylaxis rate, 56%; 95% CI, 32%-81%) in the intervention group vs 10 of 14 (71%; 95% CI, 48%-95%) in the control group (*P* = .47) (eFigure 2 in Supplement 2). Using FAAN criteria, 8 of 16 were reactors (anaphylaxis rate, 50%; 95% CI, 26%-75%) in the intervention group vs 11 of 14 (79%; 95% CI, 57%-100%) in the control group (*P* = .14) (eFigure 2 in Supplement 2). The median CoFAR score in reactors was 1.8 (IQR, 1.0-2.0) in the intervention group vs 2.6 (IQR, 2.0-3.0) in the control group (*P* = .006) (Figure 2B). The median WAO score (total range, 1-5, with higher scores indicating a more severe reaction) in reactors was 1.8 (IQR, 1.0-2.0) in the intervention group vs 2.5 (IQR, 20-3.0) in the control group (*P* = .002) (Figure 2D). There were no differences between Brighton scores or FAAN scores among reactors (eFigure 2 in Supplement 2).

**Table.** Summary of Outcome Data by Study Arm

Outcome^a^	Participants^b^		*P* value
	Intervention	Control	
Any reaction (CoFAR ≥1)	16/18 (89) [74-100]	14/22 (64) [44-84]	.08
Anaphylaxis (CoFAR ≥2) among those with any reaction	10/16 (63) [39-86]	14/14 (100) [100-100]	.02
Anaphylaxis (CoFAR ≥3) among those with any reaction	3/16 (19) [0-38]	8/14 (57) [31-83]	.06
Anaphylaxis (WAO ≥3) among those with any reaction	2/16 (13) [0-29]	8/14 (57) [31-83]	.02
Anaphylaxis likely (Brighton ≥1) among those with any reaction	9/16 (56) [32-81]	10/14 (71) [48-95]	.47
FAAN score of 1 among those with any reaction	8/16 (50) [26-75]	11/14 (79) [57-100]	.14
Anaphylaxis (CoFAR ≥2)	10/18 (56) [33-79]	14/22 (64) [44-84]	.75
Anaphylaxis (CoFAR ≥3)	3/18 (17) [0-34]	8/22 (36) [16-56]	.29
Anaphylaxis likely (Brighton ≥1)	9/18 (50) [27-73]	10/22 (45) [25-66]	.99
FAAN score of 1	8/18 (44) [21-67]	11/22 (50) [29-71]	.76
CoFAR score among those with any reaction, median (IQR)	1.8 (1.0-2.0)	2.6 (2.0-3.0)	.006
WAO score among those with any reaction, median (IQR)	1.8 (1.0-2.0)	2.5 (2.0-3.0)	.002
Time to symptom onset among reactors, median (IQR), min	7.5 (3.0-23.5)	17.5 (10.0-23.0)	.21
Any treatment administered	16/16 (100) [100-100]	14/14 (100) [100-100]	.99
Treated with epinephrine	8/16 (50) [26-74]	12/14 (86) [67-100]	.06

Abbreviations: CoFAR, Consortium for Food Allergy Research; FAAN, Food Allergy and Anaphylaxis Network; WAO, World Allergy Organization.

^a^ CoFAR score range, 1 to 5, with 2 or higher indicating anaphylaxis. WAO score range, 1 to 5, with higher scores indicating more severe reactions. Brighton score, 0 or ≥1, with 0 indicating insufficient information to categorize as anaphylaxis or sufficient to categorize as not anaphylaxis and ≥1 indicating any likelihood of anaphylaxis. FAAN score, 0 or 1, with 0 indicating no anaphylaxis and 1 indicating anaphylaxis.

^b^ Data are presented as number out of total number (percentage) of participants [95% CI] unless otherwise indicated.

None of the differences in the Table remained significant in the worst-case scenario analysis. For example, for anaphylaxis among those with any reaction (CoFAR score ≥2), we observed a difference of 63% (95% CI, 39%-86%) in the intervention group vs 100% (95% CI, 100%-100%) in the control group (*P* = .02). In our worst-case scenario analysis, this was 67% (95% CI, 45%-88%) vs 88% (95% CI, 71%-100%) (*P* = .23). eFigure 3 in Supplement 2 shows anaphylaxis rates in all participants.

**Figure 3.**
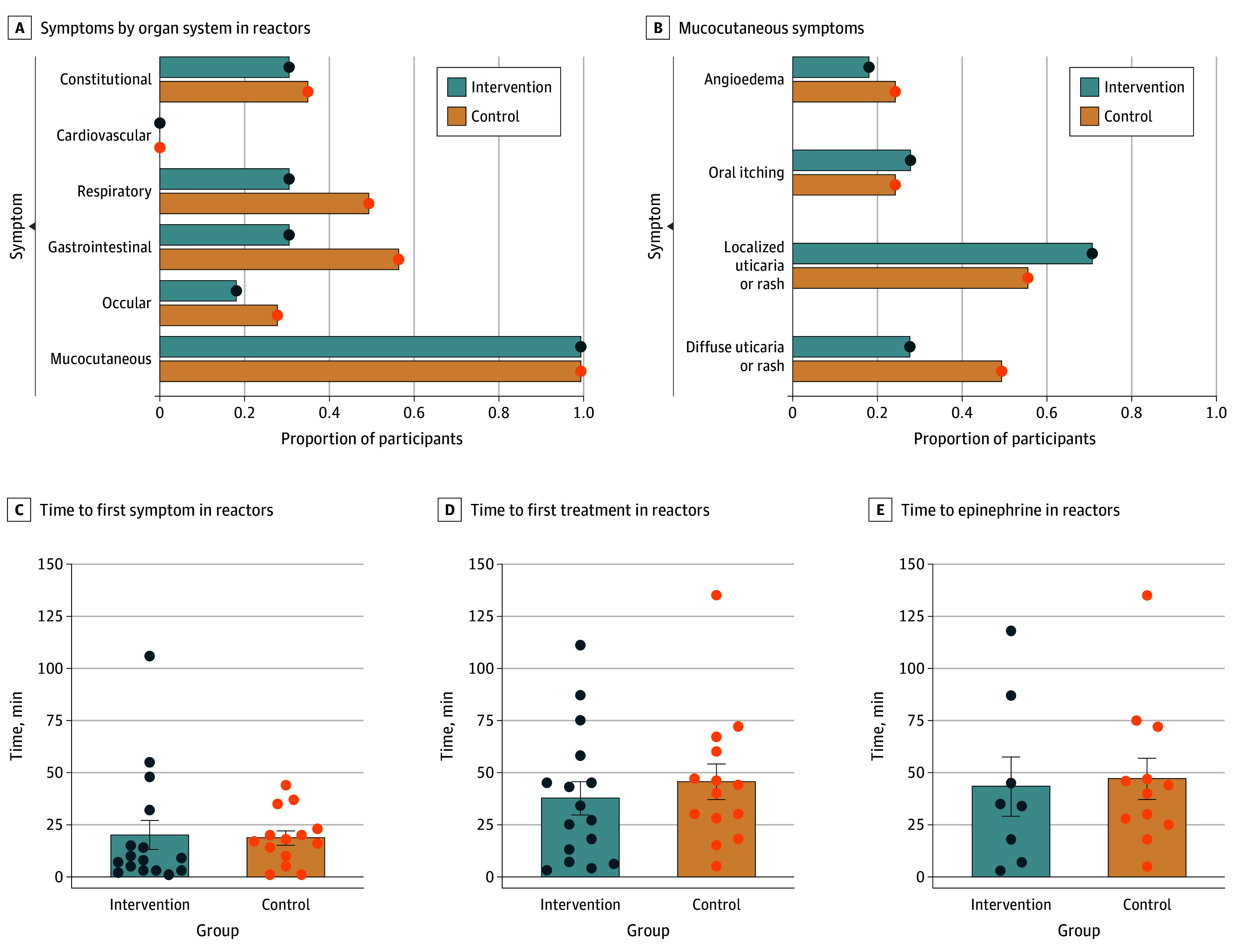
Demonstrations of Type and Timing of Symptoms Experienced by Reactors Statistical analysis used unpaired *t* tests. Error bars signify SEMs and circles, individual data points.

There were 30 reactors (75%), 16 (89%) in the intervention group and 14 (64%) in the control group (*P* = .08) (Figure 2E). All reactors in both groups received some form of pharmacologic treatment. Eight of 16 participants in the intervention group (50%; 95% CI, 26%-74%) required epinephrine vs 12 of 14 participants in the control group (86%; 95% CI, 67%-100%) (*P* = .06) (Figure 2F). We categorized reactors by organ system affected (Figure 3A). All reactors in both groups had mucocutaneous symptoms, as shown in Figure 3B. Reactors in the control group were more likely to experience respiratory, gastrointestinal, and ocular symptoms, though the study was not powered to detect statistical significance.

There was no difference in the time to first symptom onset between groups, with a mean (SD) of 18.3 (29.4) minutes in the intervention group and 18.6 (13.0) minutes in the control group (*P* = .21) (Figure 3C). There was no difference in the time to first treatment between groups, with a mean (SD) of 37.6 (31.9) minutes in the intervention group and 45.5 (32.3) minutes in the control group (*P* = .35) (Figure 3D). There was no difference in the time to epinephrine treatment between groups, with a mean (SD) of 43.4 (40.1) minutes in the intervention group and 47.1 (34.3) minutes in the control group (*P* = .62) (Figure 3E).

To better visualize TEWL trends during OFC, we created several TEWL tracing graphs for representative participants. eFigure 4 in Supplement 2 demonstrates a representative TEWL tracing in 4 participants: a nonreactor, a reactor in the intervention group who met stopping criteria, a reactor in the control group who met stopping criteria but in whom the challenge was not ended as the participant was in the control group, and a reactor who had a TEWL rise but did not qualify to engage stopping criteria as the rise occurred outside of the stipulated window.

**Figure 4.**
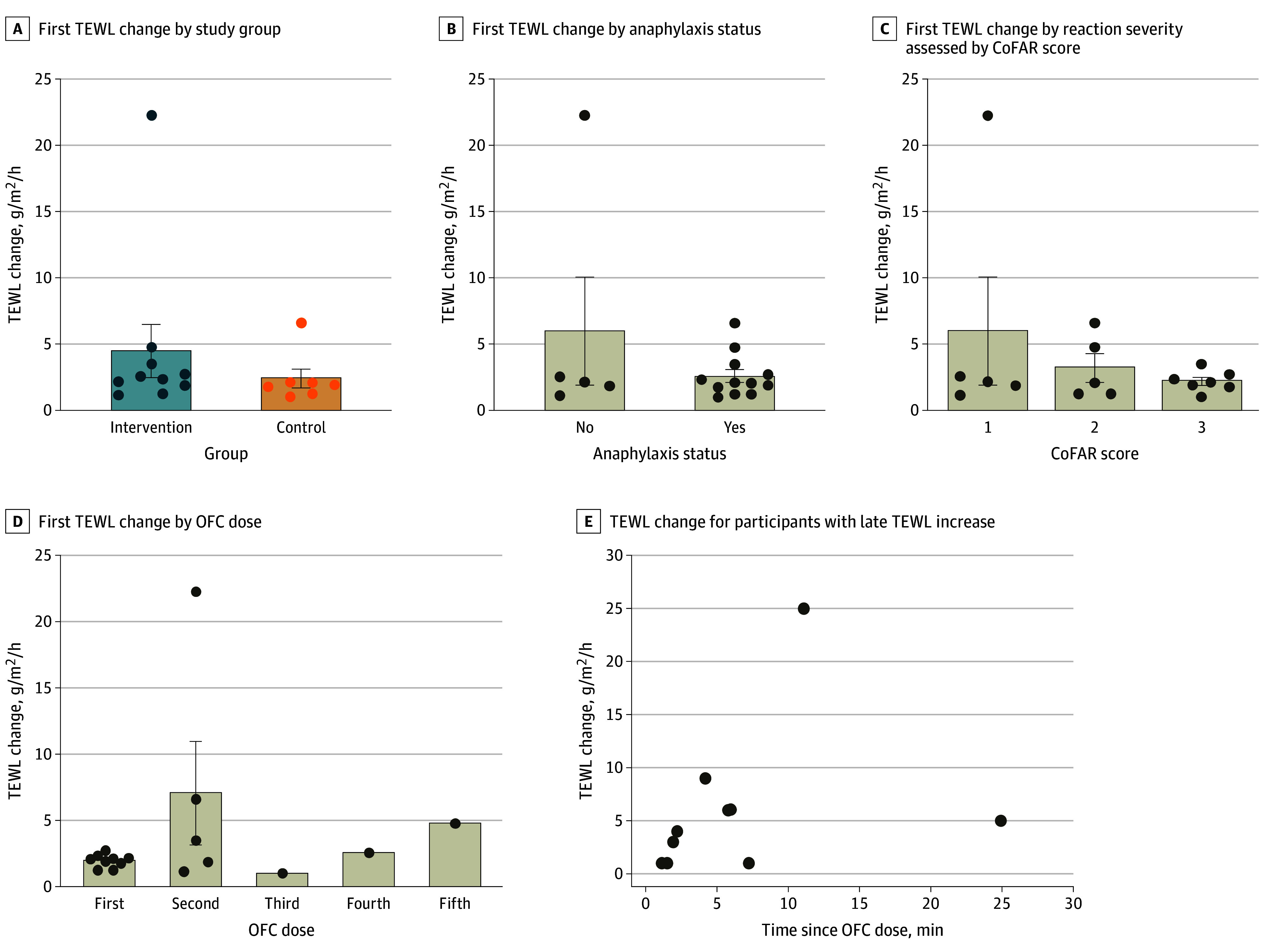
Transepidermal Water Loss (TEWL) Changes According to Various Characteristics E, TEWL change that occurred for reactors whose TEWL rise did not occur within 2 minutes of an oral food challenge (OFC) dose are shown. Statistical analysis used unpaired *t* tests and 1-way analysis of variance. CoFAR indicates Consortium for Food Allergy Research. Error bars signify SEMs and circles, individual data points.

We next examined the magnitude of TEWL rise based on intervention group, anaphylaxis status, and reaction severity. Among participants with a qualifying TEWL rise, there was no significant difference in TEWL change based on intervention group, with a mean TEWL rise of 4.45 (95% CI, −0.09 to 9.00) g/m^2^/h in the intervention group vs 2.38 (95% CI, 0.62 to 4.15) g/m^2^/h in the control group (*P* = .42) (Figure 4A). There was no significant difference in TEWL change based on anaphylaxis status, with participants experiencing anaphylaxis regardless of intervention group having a mean TEWL rise of 2.60 (95% CI, −5.32 to 17.31) g/m^2^/h vs those not experiencing anaphylaxis having a mean TEWL rise of 5.99 (95% CI, 1.56 to 3.64) g/m^2^/h (*P* = .21) (Figure 4B). There was no significant difference based on anaphylaxis severity using CoFAR scores (*P* = .45) (Figure 4C).

We examined participants who reacted but did not qualify (intervention group) or would not have qualified (control group) for stopping criteria due to not exhibiting a TEWL rise. In those 10 participants (25%), 6 (60%) had a TEWL rise occurring within 5 minutes after the challenge dose and 8 (80%) had a TEWL rise occurring within 10 minutes after the challenge dose (Figure 4E).

## Discussion

This pilot clinical trial demonstrated that TEWL measurement can be deployed effectively in young children to serve as an early warning of food anaphylaxis. In the intervention group, the reaction severity was lower than in the control group, suggesting that early intervention based on TEWL results may improve OFC safety outcomes without reducing OFC accuracy. These findings show enough promise to merit follow-up in a larger study for confirmation in a broader population.

The primary outcome in our study was a comparison of anaphylaxis rates between the intervention and control groups. We did see a significant difference between the intervention and control groups in anaphylaxis rates in participants who experienced any reaction using CoFAR and WAO criteria. However, we did not see a significant difference when using Brighton or FAAN criteria. This is reflected in the anaphylaxis rates defined by each scoring system. For example, in the control group, there was an anaphylaxis rate of 100% using CoFAR but only 71% using Brighton and 57% using WAO. This discrepancy might be attributable to how each scoring system was developed. The Brighton Collaboration criteria were originally devised for vaccine reactions^22^ and FAAN criteria to determine anaphylaxis likelihood in the emergency department setting,^23^ whereas CoFAR criteria were devised primarily to stratify food reaction severity.^21^ The WAO criteria were formatted to align with CoFAR criteria.^24^ Forming a standardized reaction and anaphylaxis assessment model remains a limitation in allergy research generally.^25^

In addition to a significant decrease in anaphylaxis rates among reactors in the intervention group, we saw a significant difference in reaction severity scoring between groups by CoFAR and WAO scores. Thus, not only were participants in the control group more likely to experience a multiorgan system reaction but they also experienced more severe reactions. This corresponded to the fact that those in the control group were more likely to require epinephrine treatment (though not all participants who met CoFAR criteria for anaphylaxis required epinephrine). Given the particular relevance of the CoFAR criteria for food reaction severity and the significant decrease in CoFAR score in the intervention group, it is likely that the CoFAR-based anaphylaxis score represents a true decrease in the severity of reactions in the intervention group in this study.

We did not see a significant difference in the time to symptoms, time to treatment, or time to epinephrine between groups. This implies that the difference in reaction severity is likely due to implementation of the TEWL stopping rule and subsequent withholding of the next food dose as opposed to faster treatment with epinephrine. Similarly, there were no significant differences in magnitude of TEWL change based on intervention group, anaphylaxis status, or anaphylaxis severity. This was important to demonstrate as it verifies that TEWL change was not dependent on these factors and that TEWL rise was equivalent between intervention and control groups. Thus, the differences seen in anaphylaxis rates and anaphylaxis severity were instead likely causally related to the primary intervention (ie, stopping criteria engagement).

Of note, it appeared that most of the TEWL increases occurred within the first 2 challenge doses (Figure 4D). This indicates that TEWL rise is an early function of the reaction timeline or physiology as opposed to a later one, which could make it an appealing tool as a reaction predictor. It is important to emphasize that TEWL rise appears to be an early indicator of anaphylaxis. This contrasts with vital-sign changes in anaphylaxis, which are often lagging rather than leading indicators.^26^ Other attempts to predict anaphylaxis previously investigated include heart rate variability,^27^ foot temperature,^28^ and facial thermography.^29^ However, there is variability in clinical significance of lead time and device accessibility with these methods.

It was important to examine why some participants did not trigger TEWL stopping criteria. We found that in those participants, a TEWL rise still occurred; however, it was most often just outside the predetermined observation window of 2 minutes (Figure 4E). As previously stated, this observation window was established based on a previous observational study of when most TEWL rises occur,^14^ though the results of this prospective pilot study indicate this window may need to be lengthened in future studies.

Although this study was not powered to detect a statistical difference in the types of symptoms reactors experienced, it is worth noting a trend toward more respiratory, gastrointestinal, and ocular symptoms in the control group. Respiratory and cardiovascular symptoms are generally considered more emergent in FA reactions; thus, preventing these specific symptoms would be helpful. Additionally, all reactors in both groups experienced at least 1 mucocutaneous symptom. Previous studies have shown that younger children are more likely to experience mucocutaneous symptoms vs older children and adults.^30,31^ These symptoms are often deemphasized in anaphylaxis grading criteria, so these findings underscore the need for standardized grading criteria for food reactions in this age group.

It is important to note that our overall reaction rate was 75%, despite the fact that entry criteria required both a clinical history of peanut reaction and positive serological and/or skin prick testing. This mirrors a common refrain in the FA field for the need for more reliable diagnostic modalities.^32^ Cutoff values for skin prick and serum IgE testing continue to vary widely in published studies, and the negative predictive value of current testing methods can be as low as 20%.^33^ This highlights the lack of reliable diagnostic ability in the field of food anaphylaxis and the need for more accurate evaluation modalities. It also highlights the current reliance on OFC for definitive determination of an FA diagnosis.^32^ Thus, anything that can make the OFC safer, more reliable, and more accessible is worth exploring. Using TEWL as an additional data point to anticipate reactions could help practitioners guide decision-making.

As this was a pilot study, a major aspect of assessment was the feasibility and practicality of our intervention, namely requiring the TEWL device to remain in place during the duration of the OFC. This was not self-evident given the young age of the participants. However, all participants were able to keep the TEWL device in place during the OFC, and measurements were reliable and accurate.

Regarding practical implementation of TEWL in a research or clinical setting, we emphasize the exploratory nature of this randomized clinical trial. Our data support an objective finding to provide decision support in an OFC, regardless of the setting. Furthermore, variation persists in when and how practitioners conduct OFCs.^34^ For example, one practitioner may stop an OFC for isolated perioral hives, while another practitioner would continue. This is affected by individual patient and challenge-food characteristics. We hope that an objective marker such as TEWL can guide the practitioner in such scenarios about next steps during that challenge.

The mechanism for how TEWL change may predict anaphylaxis is unclear. It is known that anaphylaxis clearly leads to changes in the skin, though it is unclear which specific changes are key to explaining TEWL increase. One potential mechanism is that cutaneous blood flow increases in anaphylaxis^35^ and anaphylaxis is associated with strong peripheral vasodilation.^36^ Another potential mechanism is that histamine, a key mediator in anaphylaxis, has a direct adverse effect on vascular endothelial integrity.^37,38^ Independent from histamine, other mast cell granule contents have a direct antagonistic effect on cell adhesion molecules.^39^ An acute, localized release of mast cell granule contents could thus dynamically affect the skin barrier and lead to an increase in TEWL. In either case, these phenomena merit further study.

### Limitations

This study has limitations. This was a single-center pilot study in a confined age group using a single food. Our sample size made the worst-case scenario analysis nonsignificant. Generalizability outside of this population is limited without further study. There was a smaller peanut skin wheal in our intervention group vs our control group, likely due to random chance. However, there was a higher rate of any reaction in the intervention group (89%) vs the control group (64%), and skin prick testing can help predict likelihood of reaction but not reaction severity.^40^ There were also limitations regarding the TEWL device used in this study. It was corded and had a user interface not optimized for clinical use. We are developing a wireless, clinically focused device to mitigate these shortcomings.

## Conclusions

This randomized clinical trial found that TEWL rose dynamically during peanut food reactions and may serve as a stopping criterion to decrease anaphylaxis rates and epinephrine use during OFCs. Data collection in a young population is feasible. Further work is needed to understand the mechanism for the rise in TEWL during food reactions. Replicating this study in a larger, multicenter population is needed for future development of an anaphylaxis warning system.

## Notes

Supplement 1.
Trial Protocol

Supplement 2.
eMethods.
**eFigure 1.** Schematic of Study Design **eFigure 2.** Anaphylaxis Rates and Mean Scores in Nonsignificant Scoring Systems **eFigure 3.** Anaphylaxis Rates and Epinephrine Use in All Participants (Both Reactors and Nonreactors) by Intervention Group **eFigure 4.** Representative TEWL Tracings in Select Model Participants **eTable.** Summary of Baseline Participant Characteristics by Study Arm

Supplement 3.
Data Sharing Statement
