# Supplementary material for: Transepidermal Water Loss in Oral Food Challenges in Children With Peanut Allergy: A Randomized Clinical Trial
Source: JAMA Netw Open. 2025 Nov 14;8(11):e2543371. doi: 10.1001/jamanetworkopen.2025.43371 (PMC12619095; doi:10.1001/jamanetworkopen.2025.43371)
Supplement: Supplement 1. — Trial Protocol [file jamanetwopen-e2543371-s001.pdf]

Title: Transepidermal Water Loss as a Predictor for Severe Allergic Reactions in Oral Food Challenges

Short Title: Predicting peanut anaphylaxis and reducing epinephrine

Acronym: PrePARE

Protocol Number: HUM00205852

National Clinical Trial (NCT) Identified Number: Pending

Principal Investigator:
Charles F. Schuler, MD; Allergy and Immunology and Food Allergy Center

Co-Investigators:
Kelly M. O'Shea, MD; Allergy and Immunology and Food Allergy Center
George Freigeh, MD: Allergy and Immunology

Sponsor: The University of Michigan

Funded By: Gerber Foundation

Version Number: 2.2

30 August 2023

Statement of Compliance

The trial will be carried out in accordance with International Conference on Harmonization Good Clinical Practice (ICH GCP) and the following:

- United States (US) Code of Federal Regulations (CFR) applicable to clinical studies (45 CFR Part 46, 21 CFR Part 50, 21 CFR Part 56, 21 CFR Part 312, and/or 21 CFR Part 812)

Investigators and clinical trial site staff who are responsible for the conduct, management, or oversight of clinical trials have completed Human Subjects Protection and ICH GCP Training.

The protocol, informed consent form(s), recruitment materials, and all participant materials will be submitted to the Institutional Review Board (IRB) for review and approval. Approval of both the protocol and the consent form must be obtained before any participant is enrolled. Any amendment to the protocol will require review and approval by the IRB before the changes are implemented to the study. In addition, all changes to the consent form will be IRB-approved; a determination will be made regarding whether a new consent needs to be obtained from participants who provided consent, using a previously approved consent form.

Protocol Summary

Synopsis

**Title:**	Transepidermal Water Loss as a Predictor for Severe Allergic Reactions in Oral Food Challenges
**Study Description:**	This is a pilot clinical trial, which will test the hypothesis that transepidermal water loss (TEWL) measured continuously during an oral food challenge (OFC) for peanut food allergy predicts the development of anaphylaxis such that stopping the food challenge early based on this change will reduce the rate of anaphylaxis during such food challenges.
**Objectives:**	Primary Objective: *Define anaphylaxis rates in OFCs for peanut allergy halted by TEWL stopping rules plus standard of care clinical assessment versus control OFCs, where challenges are halted based only on clinical assessment of reaction status per standard of care.*<br><br>Secondary Objectives:<br>1. Define overall allergic reaction rates in peanut OFCs halted by TEWL stopping rules versus usual care.<br>2. Define anaphylaxis likelihood and severity via Brighton and CoFAR criteria among anaphylaxis reactions in peanut OFCs halted by TEWL stopping rules versus usual care.
**Endpoints:**	Primary Endpoint: Anaphylaxis occurrence rate<br><br>Secondary Endpoints: Secondary endpoints will include anaphylaxis severity and overall allergic reaction rates.<br><br>Anaphylaxis likelihood will be defined by the Brighton criteria. Anaphylaxis severity will be defined via the modified CoFAR criteria (these are defined in the protocol).
**Study Population:**	The total study population will be 40 individuals aged 6 months to 5 years, any gender, any demographic group, with a high likelihood of peanut allergy (defined in protocol). The study will take place in Michigan.
**Phase:**	N/A
**Description of Sites/Facilities Enrolling Participants:**	This will be a single center study at the University of Michigan in Ann Arbor, MI.

**Description of Study Intervention:**	This study will evaluate the use of transepidermal water loss (TEWL) continuous measurement during oral food challenge for food allergy (peanut). A stopping rule based on TEWL will be tested against usual oral food challenge care. In both cases, the standard of care will be used, where even a single symptom of an allergic reaction is used to stop oral food challenges. TEWL measures the rate of water loss from the skin in $g/m^2/h$ as a flux of water mass through an area over time.
**Study Duration:**	24 months
**Participant Duration:**	Up to 9 days

Schema

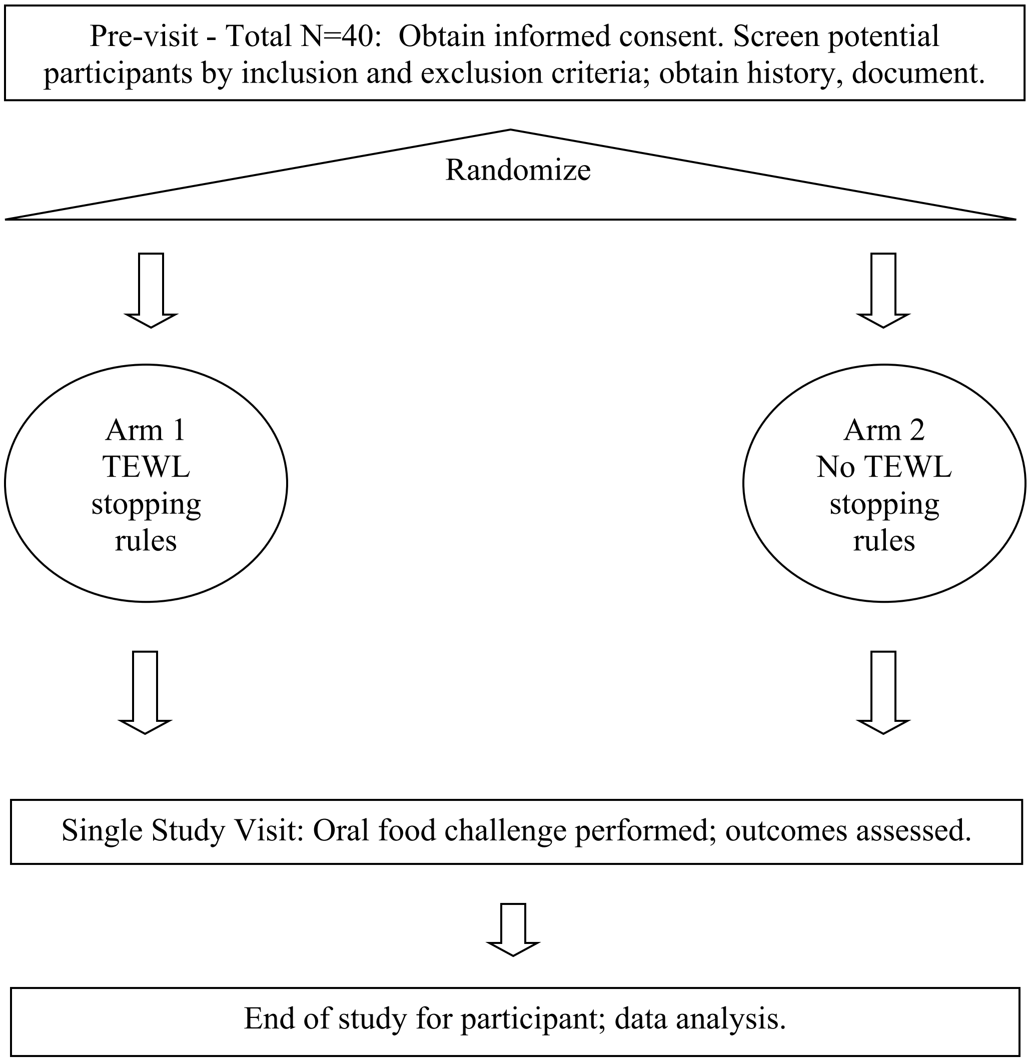
Pre-visit - Total N=40: Obtain informed consent. Screen potential participants by inclusion and exclusion criteria; obtain history, document.
Randomize
Arm 1 TEWL stopping rules
Arm 2 No TEWL stopping rules
Single Study Visit: Oral food challenge performed; outcomes assessed.
End of study for participant; data analysis.

Schedule of Activities

Procedures	Screening Day -7 to -1	OFC visit Visit 1, Day 1	Check-in call, Day 2
Pre-screening questions	X		
Informed consent	X		
Confirm eligibility	X	X	
Demographics	X		
Medical history	X		
Randomization	X		
Administer study OFC		X	
Concomitant medication review	X		
Physical exam (including height and weight)		X	
Vital signs		X	
Weight		X	
Adverse event review and evaluation	X	X	X
Complete Case Report Forms (CRFs)	X	X	X

1. Objective

Primary Objective: Define anaphylaxis rates in OFCs for peanut allergy halted by TEWL stopping rules plus standard of care clinical assessment versus control OFCs, where challenges are halted based only on clinical assessment of reaction status per standard of care.

Secondary Objectives:
1. Define overall allergic reaction rates in peanut OFCs halted by TEWL stopping rules versus usual care.
2. Define anaphylaxis likelihood and severity via Brighton and CoFAR criteria among anaphylaxis reactions in peanut OFCs halted by TEWL stopping rules versus usual care.

2. Specific Aims

Aim 1: Determine the ability of TEWL-based stopping rules to impact OFC safety features for peanut OFCs.

Sub-aim 1: Define anaphylaxis occurrence rate in OFCs halted by TEWL stopping rules versus usual care OFCs, where challenges are halted based soley on clinical assessment of reaction status.
Sub-aim 2: Define anaphylaxis likelihood and severity via Brighton and CoFAR criteria among anaphylaxis reactions in OFCs halted by TEWL stopping rules versus usual care.
Sub-aim 3: Define overall allergic reaction rates in OFCs halted by TEWL stopping rules versus usual care.

Table 1

OBJECTIVES	ENDPOINTS	JUSTIFICATION FOR ENDPOINTS
Primary		
Define anaphylaxis rates in OFCs for peanut allergy halted by TEWL stopping rules versus usual care OFCs, where challenges are halted based on clinical assessment of reaction status.	*Anaphylaxis occurrence rate*	*Anaphylaxis is the key adverse clinical outcome of “positive” OFCs and thus a major safety issue of OFCs. If TEWL can reduce anaphylaxis rate while still maintaining overall peanut allergy diagnosis accuracy, this methodology will have utility.*
Secondary		
Define overall allergic reaction rates in peanut OFCs halted by TEWL stopping rules versus usual care.	*Secondary endpoints will include anaphylaxis severity and overall reaction rates.*	*Anaphylaxis severity is a key safety question in OFCs. If anaphylaxis severity*

OBJECTIVES	ENDPOINTS	JUSTIFICATION FOR ENDPOINTS
Define anaphylaxis likelihood and severity via Brighton and CoFAR criteria among anaphylaxis reactions in peanut OFCs halted by TEWL stopping rules versus usual care.	*Anaphylaxis likelihood will be defined by the Brighton criteria. Anaphylaxis severity will be defined via the modified CoFAR criteria (these are defined later in the protocol).*	*is impacted by TEWL stopping rules, that would be valuable information.*<br><br>*Overall allergy reaction rates are important to understand non-anaphylaxis events within OFCs. Many OFCs are stopped based on a non-anaphylactic allergic reaction (i.e., isolated hives without other symptoms) per standard of care. Such non-anaphylactic events are safer than anaphylaxis by definition, so if anaphylaxis events without TEWL stopping rules are converted to non-anaphylactic reactions, that would be a very useful data-point because we would be preserving the accuracy of the OFC via standard of care while reducing the risk by avoiding anaphylaxis.*
Tertiary/Exploratory		
Define descriptive characteristics of allergy reactions/anaphylaxis among peanut OFCs wherein TEWL stopping rules are or are not used.	*Exploratory endpoints may include but are not limited to time to first treatment, time to first symptom, epinephrine use, any medication use, and time to meeting a TEWL-based stopping rule.*	*These additional endpoints provide useful safety and descriptive features that may help define larger clinical trials with this TEWL modality in the future.*

3. Background

Food allergy (FA) presents a major societal health burden because it impacts over 5 million (8%) children in the United States (US) and grows more common every year [1-3]. FA causes food anaphylaxis, a deadly, systemic allergic reaction causing over 200,000 US emergency room visits per year [4-6]. FA also causes childhood nutritional and growth deficiencies [7-10], severe anxiety for patients and caregivers [11-13], and high healthcare and family costs due to the need for epinephrine auto-injectors, healthcare visits, and food avoidance [14-18]. An accurate diagnosis is therefore crucial to avoiding a “disaster of misdiagnosis” where the consequences of perceived FA are realized due to false positive testing in the absence of true disease [7,19].

FA diagnosis relies on clinical history. FA testing such as food-specific skin and blood immunoglobulin E (IgE) assays has high false positive rates up to 30-50% and fail to predict FA reaction severity [20-22]. The oral food challenge (OFC), where a patient ingests a potential food allergen and real-life results are observed in the allergy office, is the diagnostic criterion standard but carries a substantial risk of anaphylaxis [22,23]. During an OFC, the patient ingests a food allergen in a graduated fashion up to a total dose until a full serving is eaten without incident or until an allergic reaction, typically anaphylaxis, occurs; to be clear, anaphylaxis currently relies purely on a clinical diagnosis through physician observation as there is no monitoring device for anaphylaxis.[24] Allergists face barriers to performing OFCs, and families may decline OFCs due to anaphylaxis anxiety [25-31]. Thus, FA over-diagnosis via testing without confirmatory OFCs leads to FA anxiety, increased costs, and growth/nutrition deficits from food avoidance [7,14-19,32]. Performing OFCs gives clear benefits, including improved quality of life, an expanded and more nutritious diet, and/or clarifying a diagnosis [24,29,33,34]. However, the perceived risks and benefits of OFCs may still tilt toward OFC avoidance for many physicians and patient families; therefore, a novel method of improving OFC safety and tolerability is sorely needed.

Young children have increasing rates of FA [19,35-37]; clinical trials and related international guidelines support OFC administration even under age 1 [38,39]. In young children, the lack of expressive language delays anaphylaxis symptom detection, thereby impacting challenge cessation and reaction severity [24,40-42].

No objective monitoring device for OFC-induced anaphylaxis exists. Early treatment of anaphylaxis reduces the severity of food reactions and minimizes adverse outcomes [43-45], providing rationale for developing an OFC anaphylaxis monitoring tool. Therefore, a method of safely diagnosing *clinical* food allergy in this young age group is critical.

We propose to use the transepidermal water loss (TEWL) as a safe and specific method to monitor outcomes in OFCs in young children aged 6 months to 5 years. This age group was chosen due to the lack of expressive language, the high rate of peanut allergy, and the perceived difficulties in performing OFCs in this group. TEWL is a well-established measure of cross-sectional water loss from the skin and used in evaluating topical medications and in diagnosing dermatological conditions [46-48]. TEWL measurements have shown validity across skin tones and types, with no consistent differences noted between skin tones at rest or with perturbances of the skin [49-53]. TEWL is measured quantitatively, painlessly, and non-invasively using skin contact probes to give real time results [48,54] (Figure 1). TEWL is well-reported as a measurement among infants and young children.[55-57] Newer technologies allow for continuous measurements taken over hours [48,54]. TEWL correlates with and could predict the long-term development of FA [55-57].

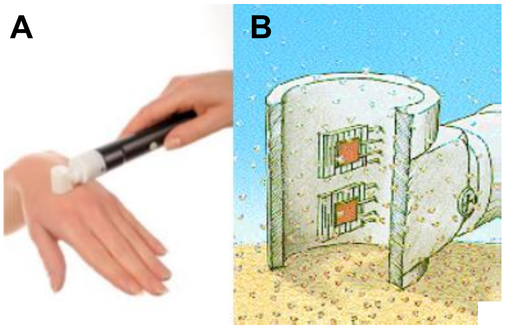

Figure 1: Transepidermal water loss is measured using a skin-contact probe placed gently upon the skin (A). Another model (see below) uses an adhesive probe head with wireless connectivity to a monitor to give continuous measurements. Inside the probe, temperature and humidity sensors measure egressing water and heat concentrations in a fixed area over time to produce the measurement (B).

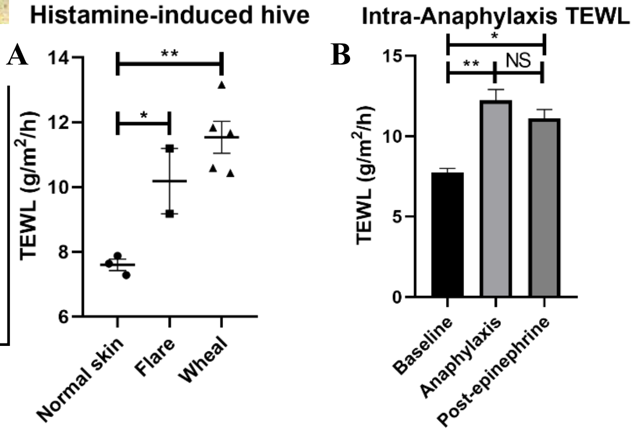

Figure 2: A) TEWL measured on a hive's wheal and flare. Measured at baseline (normal skin) and within 10 minutes after histamine applied (flare, wheal). B) TEWL measured on volar forearm during peanut-induced anaphylaxis (defined by hives and vomiting). Error bars show standard error of the mean. TEWL measured via tewameter with MPA_CTA Plus software. Means compared via ANOVA. *$p<0.05$, **$p<0.01$. NS = not significant.

TEWL in real-time anaphylaxis: TEWL has not yet been implemented in the real-time, continuous monitoring of allergic reactions or anaphylaxis. Anaphylaxis induces rapid blood vessel dilation, causing cutaneous heat and water loss.[58-62] We evaluated histamine-induced skin hives via TEWL, the positive control for skin testing.[63,64] When a hive is induced on skin via histamine, a red flare arises followed by a wheal, a pale raised area. TEWL rises over both the flare (< 1-2 minutes) and wheal (< 10 minutes) of a hive (**Figure 2A**). Most patients present with cutaneous manifestations of anaphylaxis,[65-67] lending support to evaluating continuous TEWL measurement during anaphylaxis. Furthermore, TEWL likely does rise with anaphylaxis; during a peanut-induced anaphylaxis episode with symptoms of hives and vomiting in an OFC patient, TEWL rose significantly from a baseline of 7.75 $g/m^2/h$ to 12.2 $g/m^2/h$ (**Figure 2B**), even on skin without visible hives. There was a trend toward a decrease after epinephrine. This increased TEWL does not occur in non-reactors. Since this initial observation, we have developed this technique and begun testing continuous TEWL monitoring as an anaphylaxis predictor. We have found that TEWL rises substantially in the 20 minutes after food dose 1 among anaphylaxis reactions. These data are shortly to be published.

In summary, we propose that deploying TEWL during OFCs with young children will facilitate detection of anaphylaxis prior to dangerous symptom development. We

expect that this will allow the use of OFCs with TEWL-based outcome measurements to be a definitive marker of childhood food allergy and will improve safety of the OFC. This pilot study represents a key first step in that direction.

4. Study Team Expertise

Dr. Schuler is a Clinical Assistant Professor in Allergy/Clinical Immunology, has an expertise in respiratory virus immunopathology and clinical allergy/immunology, and has served as PI on clinical data and sample collection studies as well as clinical trials involving anaphylaxis evaluation (such as for the NIH COVID-19 vaccine allergy study). He has piloted the TEWL data described and founded the UM food allergy biorepository that supported the data collection driving this work. Dr. O'Shea is a Clinical Assistant Professor in Allergy/Clinical Immunology with expertise in clinical and research-based oral food challenges. She is the PI or a CoI on multiple industry and investigator-initiated clinical trials. Dr. Freigeh holds a Master's Degree in Medical Ethics and is an allergy/immunology fellow with an interest in food allergy and ethics. He will follow along in this study (though not conduct study activities independently) as a learning exercise.

5. Methodology

a. *Inclusion/Exclusion Criteria*

Inclusion Criteria

1. Age 0.5 – 5 years as this is the time when food allergies are detected and OFCs are typically done. In addition, this timeframe is when children are least able to express symptoms they experience and are in most need of an objective anaphylaxis monitoring option.
2. Have a known history of food anaphylaxis to peanut confirmed by an allergist.
3. Have had skin and blood food allergy testing to peanut within the past 12 months. Meet the 80% likelihood positive predictive value threshold for peanut allergy based on at least 1 of either the skin or blood IgE tests per current literature corrected for age [21,68]. For peanut, this requires at least a 3 mm wheal on skin prick testing, total peanut IgE 5.0 kUa/L, and ARA H1 or H2 of > 0.35 kUa/L.
4. Meet all clinical OFC requirements. This includes no asthma or atopic dermatitis exacerbations, no recent viral infections, no recent antibiotics, and no food allergy reactions in the past month. (See appendix for protocol).

Exclusion Criteria

1. Any known cardiovascular disease, cancer, pulmonary disease except well-controlled asthma, or other condition that would preclude an OFC otherwise.
2. Any medication use that would interfere with an OFC result. Medications in this category include antihistamines (first or second generation) within 1 week, omalizumab within 3 months, and others listed in the appendix.
3. Any skin condition aside from well-controlled eczema that might impact TEWL measurement, including such conditions as

autoimmune skin conditions (such as psoriasis), congenital ichthyoses, hyper-IgE syndromes.

b. Recruitment Plan and Study Design

i. Number of Subjects

N=40 individuals

ii. Method of Contact

We will screen and recruit individuals that meet the above enrollment criteria from our food allergy clinic visits for peanut food allergy. Individuals' parents/guardians will be contacted initially by a member of the study team in clinic, over the phone, and/or email. A posting on UMHealthResearch.org will also be created.

iii. Method of Consent

Individuals' parents/guardians will be contacted as above. Individuals' parents/guardians approached for the study will be provided the consent document for review and offered background on the study. This process may take place in person during a clinical visit, over the phone, or via video (such as Zoom) by a member of the study team. Eligible individuals' parents/guardians will be provided the informed consent document and a thorough review of the document will take place. Participant parent/guardians will have as much time as needed to ask questions and decide if they would like to participate in the study. In most cases, consent will be provided to the participant electronically and they will be asked to sign using the University of Michigan's FDA 21 CFR Part 11 compliant e-signature service ("SignNow"). This service automatically provides a copy of the fully signed document to all parties and a copy will be placed in the participant's medical record. There may be circumstances where a participant is able to complete the consent process in person during a clinical visit; sufficient time for review and questions will be provided for in person consenting as well. Rarely, consents may send via postal mail for ink signatures. In any case no study procedures will take place until the consent has been signed by all parties and a copy of the fully signed document is provided to the participant. The method, location, and individuals involved in the consent process will be documented in the study records.

iv. Method of Interaction/Procedure/Intervention

Pre-OFC Visit/Interaction

1. Collect demographics from the participant and/or electronic medical record.
2. Review medical history and prior peanut food allergy testing with participant and within the electronic medical record by a study physician.
3. Assessment of concomitant medications.
4. Obtain written informed consent.
5. Participant will then be scheduled for an OFC visit

Strategies for Recruitment and Retention

We have allowed for multiple methods of contact as above to ensure that individuals with different levels of digital connectedness and

socioeconomic status can be included. In addition, we will compensate participants $100 total to participate in this study for time, travel, and parking. $50 will be provided based on arrival to the study site (to compensate for screening time, travel and parking) and the other $50 will be provided based on finishing the challenge protocol (to compensate for time spent during the challenge).

Given the pilot nature of this study the population sampled will likely represent a convenience sample. Our food allergy patients are evenly split by sex, and our population represents Washtenaw County and southeast Michigan ethnically and racially. We expect a reasonably diverse sample for this pilot study, though the lower enrollment number (n=40 total) means there is a possibility of randomly asymmetric enrollment across groups. Ultimately, ensuring a diverse sample will be even more feasible once we move to broader ages and (hopefully) multi-site studies after this pilot study.

Research OFC procedure.

OFCs will be conducted by a food allergy OFC-trained nurse; Bridgette Kaul, RN, is a member of our food allergy research team and has conducted numerous clinical OFCs. OFCs will be conducted exactly according to the clinical OFC parameters (see appendix) and final allergy diagnosis will be made per standard of care. Briefly, individuals will come to the clinical research area. They will have vital signs (height, weight, temperature, heart rate, respiratory rate, blood pressure, and SpO2 on room air) and a review of any additional symptoms or new medications since screening. The TEWL monitor will be applied to the volar forearm and monitoring initiated. Food doses will be given in a graduated fashion up to a defined single serving for the food in question and the age/size of the participant (see appendix table for food dosing). The OFC will proceed until a full dose is consumed without symptoms or a stopping criterion is reached (below). Most OFCs require 2-4 hours. Typically, 1-2 hours is required for intake and food ingestion, followed by 1-2 hours of monitoring and/or treating reactions that arise.

This will be an open OFC, meaning participants will know they are eating a likely reactive food. To be clear, an alternative approach is to perform double-blinded, placebo-controlled (DBPC) OFCs; this means the participant does two OFCs in which they receive placebo and the food in question in two separate challenges (usually a day to a week apart) and are blinded to which is done on each day. Blinding food compounds are used to obscure the food, such as applesauce or mint chocolate. While the DBPC OFC is considered the most definitive OFC method, the open OFC is considered an appropriate criterion standard and is in fact the method used clinically across the US [69,70]. Thus, given the markedly increased costs of DBPC OFCs and the lack of their use in FA clinics, we will to use the open OFC in this pilot study.

Blinding procedures.

Participants and families, the food allergy nurse administering the OFC, and the allergist physician assessing and treating the patient will be blinded to the study arm status (i.e., TEWL stopping rule intervention group

or control group). The study coordinator monitoring TEWL during the OFC will need to know the intervention arm vs control arm status to declare the OFC ceased when stopping rules are reached; this person will have no say in assessment or treatment of reactions. The study coordinator monitoring TEWL will be in the same room as the participant but will be behind a screen so that the screen displaying the TEWL results and the study coordinator's face will not be immediately visible to the other team members. All subjects will have TEWL monitored throughout to control for confounding factors associated with having a monitoring device on the skin. At the end of the OFC, after the determination of OFC outcomes, the participants and families will be made aware of the overall OFC determination of allergy status.

Regardless of group assignment (TEWL stopping rule or usual care with TEWL simply measured but not reported during the OFC), we expect that participants will be able to use the results of the OFC to reliably define true clinical food allergy status. Diagnosis will rely on standard of care in both cases.

Randomization.

Participants will be randomized 1:1 to the intervention and control groups. We will use permuted block randomization. We will vary the block size from 2 to 4 in order to enhance blinding. We will upload a 40-person participant list to randomization software that will produce the randomization, which will be performed at visit 1 (initial screening visit) or no later than the day of the research food challenge. The TEWL study coordinator will learn the group designation for each participant at the start of that participant's challenge by the software.

TEWL measurement methods.

All subjects will undergo continuous TEWL measurement [48,54]. We will use a Tewameter VT310 device (Khazaka, Germany); these probes and supporting software were supplied at no cost and with no obligation except mention of material support in publications by the manufacturer. Measurements are taken using a small adhesive to attach the probe to the skin. Measurements are taken on the volar forearm. Data are collected in MPA CT Plus software (Khazaka, Germany) which provides note-taking functions and exports data into Microsoft Excel (Seattle, WA) for analysis.

Adjudication of OFC results.

A FA physician will adjudicate reactions and diagnose anaphylaxis identically to usual care in the FA clinic. The Brighton anaphylaxis likelihood scale will be used to assess anaphylaxis likelihood. The decision tree is copied here (next page, Figure 3). The CoFAR anaphylaxis severity scale will be used for anaphylaxis severity[71] and is below in the adverse event grading section of the protocol. The Brighton score gives a 0-3 score of anaphylaxis likelihood (0 = no anaphylaxis, 3 = highly likely). The CoFAR severity scare gives a grade of 1-5 of anaphylaxis severity (where 1 = mild, 5 = death).

Any symptom typical of food allergy (see Brighton and CoFAR scales) will count as a reaction, consistent with standard of care diagnosis for peanut allergy. Note that this is distinct from anaphylaxis.

TEWL-based stopping rules will be applied to the intervention group. We plan to use a 1 $g/m^2/h$ increase in TEWL value averaged over the 2 minute period after a fully-consumed food dose versus the 2 minute period averaged over the period just prior to that food dose. If the individual meets this threshold, at the first sign of a clinical symptom, the food challenge will be stopped.

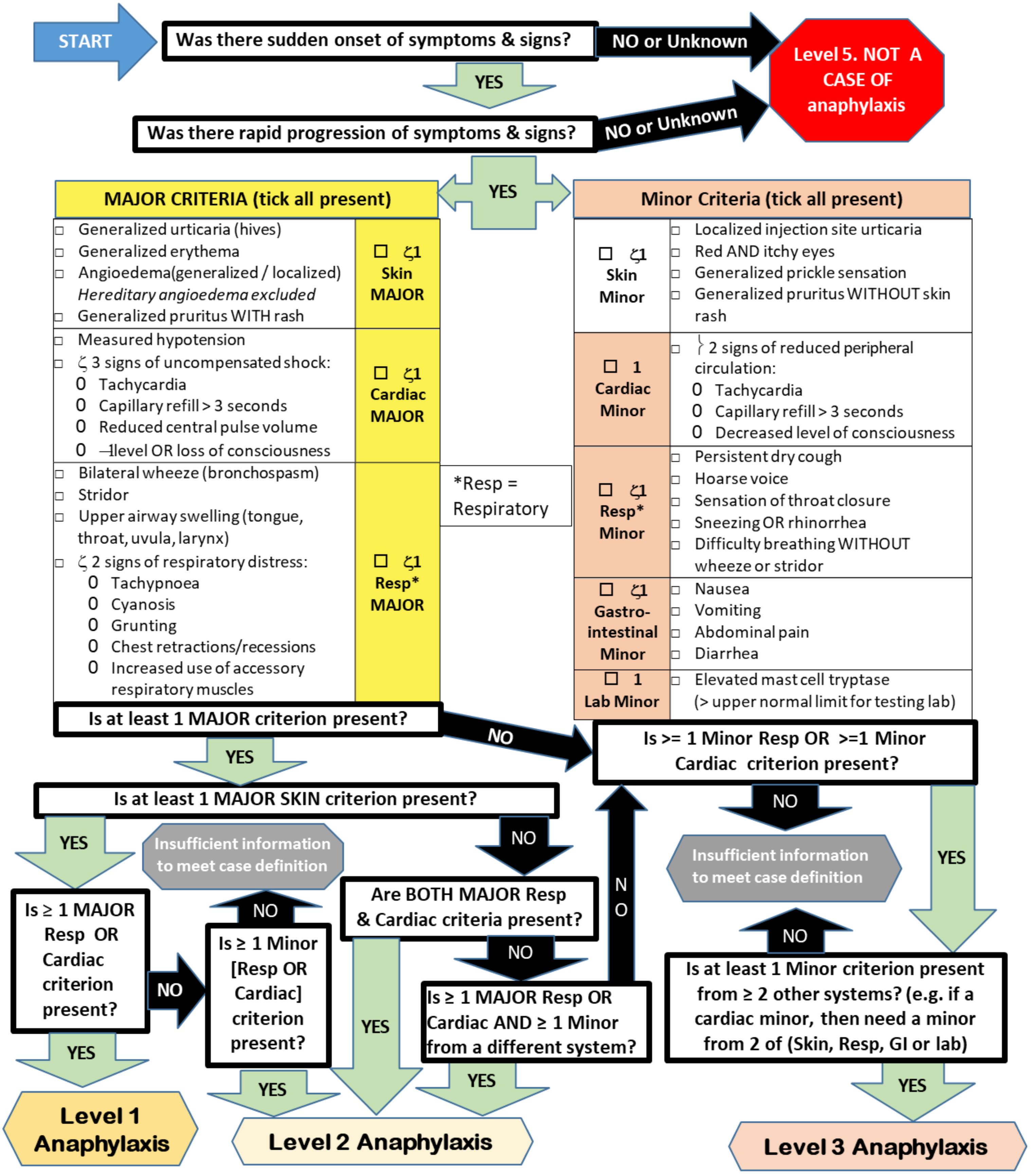

Figure 3: Brighton Anaphylaxis Likelihood Scale

Treatment of reactions.

Reaction treatment will be based on symptoms assessed by the allergist per the clinical standard of care. If the participant meets anaphylaxis criteria [71], then weight-based anaphylaxis epinephrine dosing will be given, with a follow-up dose 5 minutes later if symptoms do not resolve. Albuterol via nebulizer or inhaler/mask (depending on age) and liquid diphenhydramine and cetirizine (both are antihistamines and use weight-based dosing) will be available and used after epinephrine in the case of anaphylaxis. All doses will be calculated prior to the challenge. If a participant has a reaction not meeting anaphylaxis criteria (i.e., they only have hives but no additional symptoms), the physician may choose to stop the OFC and give an antihistamine. If the TEWL-based stopping rule is invoked, the OFC will be stopped (no more food doses given); if the participant meets anaphylaxis criteria at that time, epinephrine will be given, otherwise an antihistamine dose will be given per standard of care.

**Table 2: Outcome measures**		**Definition**
**Primary outcome**	Anaphylaxis rate	Any Brighton Level 1, 2, or 3 anaphylaxis (see table above)
**Secondary outcomes**	Reaction rate	Any objective symptom of allergic reaction occurs (e.g., hives, angioedema, vomiting, wheeze, etc) not rising to the definition of anaphylaxis.
	Anaphylaxis severity	CoFAR score, defined below
	Anaphylaxis likelihood	Brighton score, defined above
**Exploratory endpoints (Partial list)**	Time to first symptom	Time from first food dose to any symptom
	Reaction symptoms	All reaction symptoms listed
	Epinephrine use	Any epinephrine administration
	Any medication use	Any medication administration
	Time to stopping rule	Time in minutes to stopping rule trigger

Outcome measures. The outcome measures collected for each group in Table 2 show the hypothesized relationship for each group. If a stopping rule is implemented, that will be considered a positive challenge. We hypothesize that the intervention group will have lower anaphylaxis rates and/or severity via earlier OFC stoppage and earlier time to or more frequent treatment with non-epinephrine medications (such as antihistamines). We suspect the overall reaction rate will remain the same, but that these reactions will not devolve into anaphylaxis in the intervention group due to the above factors. Anaphylaxis will be defined according to current practice parameters [72], which is broadly considered to be at least two symptoms consistent with allergic reactions (cutaneous, gastrointestinal, respiratory, elevated heart rate, among others) or a decrease in blood pressure.

Post-OFC

1. Participants without evidence of an allergic reaction during the OFC will receive a follow-up phone call 1 day after the OFC to follow up on any additional symptoms or concerns.

2. Participants that had a reaction will be scheduled for a follow-up call within 1 day of the OFC to assess for additional symptoms or resolution of symptoms per the standard of care.

c. Subject Withdrawal

i. Under what conditions will a subject be withdrawn prior to completion

ii. If a subject withdraws prior to completion, what is the plan for the use of their data

Any subject may withdraw at any time for any reason. These reasons may include but are not limited to:

- Subject's request, no reason needed
- Adverse event – at Investigator's request. Potential examples include concern that proceeding further would impose unanticipated risk not otherwise delineated here or for any reason that continued participation could impact study integrity.

The data for any subject who withdraws prior to completion will be flagged as incomplete data in our data set. We will delete data if the subject requests this.

d. Data Retention and/or Data Destruction Plan

i. How long will you keep subject data?

ii. If you plan to destroy the data, how will you destroy it?

Data collected will be retained for study record keeping purposes and for future research use for up to 5 years or in accordance with our sponsor's policies. Thereafter, data will be fully de-identified fully and retained indefinitely. No data will be destroyed.

Risks & Benefits

a. What are the risks and what will be done to monitor the risks?

b. What is the likelihood of each risk (common, likely, infrequent, or rare)?

Potential Risks

- Allergic reactions, including anaphylaxis, other hypersensitivity reactions (e.g., rash, flushing, pruritus, urticaria, angioedema), diarrhea, vomiting do occur during positive OFCs. Given the goal of this study is to enroll participants who are likely to have a reaction, this is deemed LIKELY
- If a participant experiences a systemic allergic reaction, the site will provide the participant with an epinephrine auto-injector 2-pack if they do not have one. In addition, participants who experience a systemic allergic reaction will receive a Food Allergy Action Plan at the OFC visit if one does not exist. The PI or designee will review the plan including symptoms of an allergic reaction and steps to take in the event of an allergic reaction, including training on how to use the epinephrine auto-injector, with the participant prior to discharge. If a participant has an allergic reaction, he/she may need oral, IM, or IV medications. The investigators for this trial are allergists, trained to recognize,

and familiar with the treatment of anaphylaxis, and will be available within 60 seconds in the event of a reaction. Emergency medications, oxygen, and equipment will be available to treat any allergic reactions. POSSIBLE

- Treatment of individual acute allergic reactions during the conduct of the study should be with epinephrine, IV fluids, β-adrenergic agonists (e.g., albuterol), oxygen, antihistamines, and steroids, as indicated for the severity of the reaction according to the standard of care. POSSIBLE
    - Risks of these common medications are summarized below:
        - Antihistamines: drowsiness, dizziness, constipation, stomach upset, blurred vision, or dry mouth/nose/throat
        - Epinephrine: tachycardia, palpitations, nervousness, sweating, nausea, vomiting, trouble breathing, headache, dizziness, anxiety, tremors, or pale skin
        - β-adrenergic agonists: nervousness, shaking (tremor), headache, or dizziness
        - Steroids: nausea, vomiting, loss of appetite, heartburn, trouble sleeping, increased sweating, or acne
- Risks associated with TEWL measurement could conceivably include skin irritation. RARE
- Participation in this study could cause distress due to the experience of anaphylaxis. LIKELY.
- Participation in this study poses a risk for breach of confidentiality. RARE
- Taking part in more than one research study may be harmful to the subject. If subjects are already taking part in another study, we ask that they let us know. Subjects should not take part in more than one study at the same time, unless the subject and the investigators agree that the subject is not likely to be harmed, and the outcome of the study will not be disturbed. RARE
- As with any research study, though, there may be additional risks of participating that are unforeseeable or hard to predict. RARE

What will be done to reduce or monitor these risks?

- Any information sharing would only be done in the context of a new IRB review and in concert with UM's Data Management Office. To minimize the breach of confidentiality risk, information shared outside of the University of Michigan will not use the subject's name or hospital number, but rather a unique study number, and would only occur under a data use agreement supported by the University. Any publications that result from this work will only report de-identified data.
- The site investigators for this trial are allergists. They are trained to recognize symptoms and are expert in the treatment of anaphylaxis. They will be available within 60 seconds in the event of a reaction. Emergency medications, oxygen, and equipment will be available to treat any allergic reactions.
- The clinical team will provide prescriptions for epinephrine auto-injectors for participants that experience systemic allergic reactions who do not have them. In addition, participants will receive Food Allergy Action Plan at the OFC visit if they do not have one already. The PI or designee will review the plan including symptoms of an allergic reaction and steps to take in the event of an allergic reaction, including training on how to use the epinephrine auto-injector, with the participant prior to discharge.

c. What are the benefits?

iii. To the individual

Individuals and their families who undergo OFCs experience greater peace of mind and improved quality of life, even when the OFC leads to a reaction, due to improved certainty and a clarified diagnosis. Individuals and their families who pass the OFC (estimated to be 20% of both groups) will learn that they do not have a clinical food allergy, which has profound psychological, nutritional, cost, and overall health benefits, allowing the family to incorporate that food back into the diet. **Overall, we expect that individuals and their families who participate will have an accurate determination of food allergy status for the food challenged (peanut), which can be used clinically to define allergy, or the lack of allergy, for that food, per the standard of care.**

In addition, our co-investigator Kelly O'Shea, MD, who will conduct many of these research challenges, is the peanut oral immunotherapy clinic expert here at U-M. She can provide a brief discussion of the risks and benefits of OIT (which is the only FDA-approved peanut allergy preventative therapy available) for individuals who do have a peanut reaction during this clinical trial. If that therapy option is of interest, they can be streamlined into the OIT clinical practice based on the results of this OFC.

iv. To society

This data is valuable for evaluating a novel detection/prediction method for anaphylaxis. We expect results from this project to show that TEWL can detect evolving anaphylaxis and that stopping rules based on TEWL will allow OFCs to be stopped and reactions treated prior that endpoint. This would reduce the risk and potentially the cost of an OFC, thereby allowing more allergists and patients to access this critical procedure, the OFC. Furthermore, TEWL measurement may ultimately have a role in anaphylaxis measurement in additional contexts such as the ambulance, emergency room, or hospital. This is an unmet need, as anaphylaxis frequently goes unrecognized, leading to absent or delayed lifesaving epinephrine administration, even in the emergency room.

Data & Safety Monitoring

a. *Will there be a board your study will report adverse events and other problems to?*

Yes, the study will have a Data Safety and Monitoring Board (DSMB). The DSMB has a charter, which defines the membership, responsibilities, meeting schedule, and meeting substance/materials in detail. This charter is attached.

Briefly, the DSMB will meet at study initiation, every 6 months while the study is enrolling, and at least once after the final subject is enrolled and finishes study activities. All adverse events will be compiled and reported to the DSMB in written format. Any serious adverse events deemed possibly, probably, or definitely related to study procedures will be communicated to the chair of the DSMB who will decide whether the Board needs to have an interim meeting to

discuss the event. We anticipate 4-5 scheduled DSMB meetings during the 12–24-month study period.

b. *Adverse Events*

Adverse Events (AEs): Any untoward medical occurrence associated with the use of an intervention in humans, whether or not considered intervention related. AEs include expected and unexpected harmful effects, and unexpected risks of an interaction or an intervention.

Serious Adverse Event (SAE): An adverse event or suspected adverse reaction is considered “serious” if, in the view of the investigator:

1. Death.
2. A life-threatening event: An AE is considered “life-threatening” if, in the view of the investigator, its occurrence places the subject at immediate risk of death. It does not include an AE that, had it occurred in a more severe form, might have caused death. **For the purposes of this study, which is expected to include anaphylaxis during the majority of these food challenges, if the anaphylaxis event is treated during the study visit and does not require escalation to Emergency Room care, this would NOT be considered an SAE.**
3. Inpatient hospitalization.
4. Persistent or significant incapacity or substantial disruption of the ability to conduct normal life functions.
5. Congenital anomaly or birth defect.
6. Important medical events that may not result in death, be life threatening, or require hospitalization may be considered serious when, based upon appropriate medical judgment, they may jeopardize the subject and may require medical or surgical intervention to prevent one of the outcomes listed above.

The investigator must report adverse events regardless of relationship to study therapy regimen or study mandated procedures.

i. Method of Identifying, Recording, Monitoring and Reporting Adverse Events

Identifying:

For this study, an adverse event will include the following associated with the oral food challenge:

All AEs occurring within 1 day of the OFC visit.

Collecting Adverse Events: Adverse events may be discovered through any of these methods:

Observing the participant

Receiving an unsolicited complaint from the participant/family. Participants/families will have study contact information including a monitored phone number to report AEs. We will also provide advice on whether urgent medical attention is required, even though this would be expected to be a rare event.

Follow up phone call above.

Grading of Adverse Events Other than Systemic Allergic Reactions: The study sites will grade the severity of non-allergic adverse events experienced by the study participants according to the criteria set forth in the FDA Guidance for Industry Toxicity Grading Scale for Healthy Adult and Adolescent Volunteers Enrolled in Preventive Vaccine Clinical Trials (September 2007); hereafter, referred to as the FDA Toxicity Grading Scale. Adverse events will be graded on a scale from 1 to 5 according to the following standards in the FDA Toxicity Grading Scale:

Grade 1 = Mild
Grade 2 = Moderate
Grade 3 = Severe
Grade 4 = Life-threatening
Grade 5 = Death

Grading of Systemic Allergic Reactions:

The investigator will grade severity of systemic allergic reactions on a scale of 1 to 5 according to criteria set forth in the CoFAR Grading Scale.

CoFAR Grading Scale for Systemic Allergic Reactions Version 3.0:

Grade 1: Reaction involving one of the following organ systems in which the symptoms are mild:

- Cutaneous: Generalized pruritus, generalized urticaria, flushing, angioedema
- Upper respiratory: Rhinitis, cough unrelated to laryngeal edema or bronchospasm
- Conjunctival: Injection/redness, itching, tearing
- GI: Nausea, abdominal pain (no change in activity level), single episode of vomiting and/or single episode of diarrhea

Grade 2: Reaction involving two or more of the following organ systems in which the symptoms are mild:

- Cutaneous: Generalized pruritus, generalized urticaria, flushing, angioedema
- Upper respiratory: Rhinitis, cough unrelated to laryngeal edema or bronchospasm
- Conjunctival: Injection/redness, itching, tearing GI Nausea, abdominal pain (no change in activity level), single episode of vomiting, and/or single episode of diarrhea

OR

Reaction involving at least one of the following organ systems in which the symptoms are moderate:

- Cutaneous: Generalized pruritus, generalized urticaria, flushing, angioedema

- Upper respiratory: Rhinitis, cough unrelated to laryngeal edema or bronchospasm
- Conjunctival: Injection/redness, itching, tearing
- GI: Nausea, abdominal pain (with change in activity level), two episodes of vomiting and/or diarrhea

Grade 3: Reaction involving one or more of the following organ systems:

- Lower respiratory: Throat tightness, wheezing, chest tightness, dyspnea, cough that respond to short acting bronchodilator treatment (including IM epinephrine) with or without supplemental oxygen
- GI: Severe abdominal pain, more than two episodes of vomiting and/or diarrhea
- Cardiovascular: Reduced BP with lightheadedness, presyncope or tachycardia

Grade 4: Life-threatening reaction involving one or more of the following organ systems with or without other symptoms listed in Grades 1 to 3:

- Lower respiratory: Throat tightness with stridor, wheezing, chest tightness, dyspnea, or cough associated with a requirement for supplemental oxygen and refractoriness to short-acting bronchodilator treatment (including IM epinephrine)[1]
  OR
- Respiratory compromise requiring mechanical support
- Cardiovascular: Reduced BP with associated symptoms of end-organ dysfunction (e.g., hypotonia [collapse], syncope) defined as: systolic BP of less than 90 mmHg or >30% decrease from baseline

Grade 5: Death

1. Examples of refractoriness could include continuous albuterol nebulizer or epinephrine IV infusion or more than three IM epinephrine injections

The investigator will also grade likelihood of systemic allergic reactions according to criteria set forth by the Brighton Collaboration case definition and guidelines for anaphylaxis. Please see attached supporting documents in Section 44.

Recording/Monitoring:
Throughout the study, the investigator will record adverse events and serious adverse events on the appropriate AE/SAE electronic Case Report Form (eCRF) regardless of the relationship to the OFC or study procedure. Once recorded, an AE/SAE will be followed until it resolves with or without sequelae, or the AE/SAE stabilizes, or until 1 days after the participant’s OFC, whichever occurs first.

Attribution of Adverse Events Code Descriptor Relationship

RELATED CATEGORIES

1. Definitely Related
2. Probably Related
3. Possibly Related

NOT RELATED CATEGORY

4. Unlikely to be Related
5. Definitely not Related

Reporting:
We will report any adverse events and other reportable incidences and occurrences (ORIO) to the IRB. Any adverse event or ORIO will be documented of that event including a description, subject number, date, outcome, and follow-up. Reporting of adverse events and ORIOs will follow IRBMED's reporting timetable and will occur at least yearly.

Statistical Design

Stopping rules. The anaphylaxis prediction threshold used in the study 2 trial will be data-driven based on results from our ongoing data collection study. We will perform a series of logistic regression models testing the predictive value of various levels of absolute increases in TEWL, thresholds of TEWL and their combinations. These will be tested against time to first symptom, time to formal anaphylaxis diagnosis, time to first medication use, and time to epinephrine use. To avoid overfitting, these analyses will use a 2:1 derivation: validation random split. Corrections will include baseline TEWL value and variance. A minimum sustained timespan meeting the threshold (such as 30-60 seconds) may be needed to minimize the effects of noise in the measurement.

Endpoints. The primary endpoint will be anaphylaxis rate among the intervention group versus the control group and will be reported with a 95% confidence interval. Secondary endpoints will include anaphylaxis severity and overall reaction rates. Exploratory endpoints may include but are not limited to time to first treatment, time to first symptom, epinephrine use, any medication use, and time to meeting a stopping rule (regardless of whether it was implemented); all will be reported with 95% confidence intervals. Exact p-values will be calculated using Fischer's test.

Descriptive analyses. Descriptive analyses will be reported. Continuous baseline measures will be reported using either mean (or geometric mean) with 95% confidence interval or median with first and third quartiles, as appropriate. Categorical baseline and demographic characteristics and study disposition will be reported as frequencies and proportions.

Population size. To achieve 80% power with two-sided alpha=0.05 to detect a minimum 50% absolute difference in anaphylaxis rate reducing in anaphylaxis between the intervention group (anticipated anaphylaxis rate 30%) and the control group (80%) using a Fischer's exact test and at a ratio of 1:1 for both groups, 38 total subjects (19 per group) would be needed for this phase. To account for 5% drop-out, we will enroll 40 total subjects (20 per group). This is achievable with the enrollment-eligible population available to us. The Food Allergy clinics at the University of Michigan see approximately 500-1000 peanut allergy patients annually age 5 and under at the Domino Farms clinic site alone. Over 18-24 months and with a 2.5-5% rate of enrollment, enrollment of 40 participants is

feasible. There are also 3 additional Allergy clinics at U-M that could also provide a similar patient population size as well to augment this.

References

1. Gupta RS, Warren CM, Smith BM, et al. The Public Health Impact of Parent-Reported Childhood Food Allergies in the United States. *Pediatrics.* 2018;142(6).
2. Gupta RS, Warren CM, Smith BM, et al. Prevalence and Severity of Food Allergies Among US Adults. *JAMA Netw Open.* 2019;2(1):e185630.
3. Bilaver LA, Chadha AS, Doshi P, O'Dwyer L, Gupta RS. Economic burden of food allergy: A systematic review. *Ann Allergy Asthma Immunol.* 2019;122(4):373-380 e371.
4. Rudders SA, Banerji A, Vassallo MF, Clark S, Camargo CA, Jr. Trends in pediatric emergency department visits for food-induced anaphylaxis. *J Allergy Clin Immunol.* 2010;126(2):385-388.
5. Robinson LB, Arroyo AC, Faridi MK, Rudders S, Camargo CA, Jr. Trends in US Emergency Department Visits for Anaphylaxis Among Infants and Toddlers: 2006-2015. *J Allergy Clin Immunol Pract.* 2021;9(5):1931-1938 e1932.
6. Sicherer SH. Epidemiology of food allergy. *J Allergy Clin Immunol.* 2011;127(3):594-602.
7. Mehta H, Groetch M, Wang J. Growth and nutritional concerns in children with food allergy. *Curr Opin Allergy Clin Immunol.* 2013;13(3):275-279.
8. Hobbs CB, Skinner AC, Burks AW, Vickery BP. Food allergies affect growth in children. *J Allergy Clin Immunol Pract.* 2015;3(1):133-134 e131.
9. Sova C, Feuling MB, Baumler M, et al. Systematic review of nutrient intake and growth in children with multiple IgE-mediated food allergies. *Nutr Clin Pract.* 2013;28(6):669-675.
10. Mehta H, Ramesh M, Feuille E, Groetch M, Wang J. Growth comparison in children with and without food allergies in 2 different demographic populations. *J Pediatr.* 2014;165(4):842-848.
11. Dantzer JA, Keet CA. Anxiety associated with food allergy in adults and adolescents: An analysis of data from the National Health and Nutrition Examination Survey (NHANES) 2007-2010. *J Allergy Clin Immunol Pract.* 2020;8(5):1743-1746 e1745.
12. Abrams EM, Simons E, Roos L, Hurst K, Protudjer JLP. Qualitative analysis of perceived impacts on childhood food allergy on caregiver mental health and lifestyle. *Ann Allergy Asthma Immunol.* 2020;124(6):594-599.
13. DunnGalvin A, Blumchen K, Timmermans F, et al. APPEAL-1: A multiple country European survey assessing the psychosocial impact of peanut allergy. *Allergy.* 2020.
14. Dyer AA, Negris OR, Gupta RS, Bilaver LA. Food allergy: how expensive are they? *Curr Opin Allergy Clin Immunol.* 2020;20(2):188-193.
15. Gupta R, Holdford D, Bilaver L, Dyer A, Holl JL, Meltzer D. The economic impact of childhood food allergy in the United States. *JAMA Pediatr.* 2013;167(11):1026-1031.

16. Westermann-Clark E, Pepper AN, Lockey RF. Economic considerations in the treatment of systemic allergic reactions. *J Asthma Allergy.* 2018;11:153-158.
17. Shaker M, Chalil JM, Tran O, et al. Commercial claims costs related to health care resource use associated with a diagnosis of peanut allergy. *Ann Allergy Asthma Immunol.* 2020;124(4):357-365 e351.
18. Shaker M, Bean K, Verdi M. Economic evaluation of epinephrine auto-injectors for peanut allergy. *Ann Allergy Asthma Immunol.* 2017;119(2):160-163.
19. Sicherer SH, Sampson HA. Food allergy: A review and update on epidemiology, pathogenesis, diagnosis, prevention, and management. *J Allergy Clin Immunol.* 2018;141(1):41-58.
20. Koplin JJ, Perrett KP, Sampson HA. Diagnosing Peanut Allergy with Fewer Oral Food Challenges. *J Allergy Clin Immunol Pract.* 2019;7(2):375-380.
21. Peters RL, Allen KJ, Dharmage SC, et al. Skin prick test responses and allergen-specific IgE levels as predictors of peanut, egg, and sesame allergy in infants. *J Allergy Clin Immunol.* 2013;132(4):874-880.
22. Kawahara T, Tezuka J, Ninomiya T, et al. Risk prediction of severe reaction to oral challenge test of cow's milk. *Eur J Pediatr.* 2019;178(2):181-188.
23. Calvani M, Berti I, Fiocchi A, et al. Oral food challenge: safety, adherence to guidelines and predictive value of skin prick testing. *Pediatr Allergy Immunol.* 2012;23(8):755-761.
24. Bird JA, Leonard S, Groetch M, et al. Conducting an Oral Food Challenge: An Update to the 2009 Adverse Reactions to Foods Committee Work Group Report. *J Allergy Clin Immunol Pract.* 2020;8(1):75-90 e17.
25. Herbert LJ, Dahlquist LM, Bollinger ME. Maternal intolerance of uncertainty, anxiety, and adherence with food challenge referrals. *J Health Psychol.* 2013;18(9):1209-1219.
26. Strinnholm A, Brulin C, Lindh V. Experiences of double-blind, placebo-controlled food challenges (DBPCFC): a qualitative analysis of mothers' experiences. *J Child Health Care.* 2010;14(2):179-188.
27. Weiss D, Marsac ML. Coping and posttraumatic stress symptoms in children with food allergies. *Ann Allergy Asthma Immunol.* 2016;117(5):561-562.
28. Greiwe J, Oppenheimer J, Bird JA, et al. AAAAI Work Group Report: Trends in Oral Food Challenge Practices Among Allergists in the United States. *J Allergy Clin Immunol Pract.* 2020;8(10):3348-3355.
29. Knibb RC, Ibrahim NF, Stiefel G, et al. The psychological impact of diagnostic food challenges to confirm the resolution of peanut or tree nut allergy. *Clin Exp Allergy.* 2012;42(3):451-459.
30. Hsu E, Soller L, Abrams EM, Protudjer JLP, Mill C, Chan ES. Oral Food Challenge Implementation: The First Mixed-Methods Study Exploring Barriers and Solutions. *J Allergy Clin Immunol Pract.* 2020;8(1):149-156 e141.
31. Byrne AM, Trujillo J, Fitzsimons J, et al. Mass food challenges in a vacant COVID-19 stepdown facility: exceptional opportunity provides a model for the future. *Pediatr Allergy Immunol.* 2021.
32. Alsaggaf A, Murphy J, Leibel S. Estimating Cost-Effectiveness of Confirmatory Oral Food Challenges in the Diagnosis of Children With Food Allergy. *Glob Pediatr Health.* 2019;6:2333794X19891298.

33. Kansen HM, Le TM, Meijer Y, et al. The impact of oral food challenges for food allergy on quality of life: A systematic review. *Pediatr Allergy Immunol.* 2018;29(5):527-537.
34. Franxman TJ, Howe L, Teich E, Greenhawt MJ. Oral food challenge and food allergy quality of life in caregivers of children with food allergy. *J Allergy Clin Immunol Pract.* 2015;3(1):50-56.
35. Motosue MS, Bellolio MF, Van Houten HK, Shah ND, Campbell RL. Increasing Emergency Department Visits for Anaphylaxis, 2005-2014. *J Allergy Clin Immunol Pract.* 2017;5(1):171-175 e173.
36. Keet CA, Savage JH, Seopaul S, Peng RD, Wood RA, Matsui EC. Temporal trends and racial/ethnic disparity in self-reported pediatric food allergy in the United States. *Ann Allergy Asthma Immunol.* 2014;112(3):222-229 e223.
37. In: Oria MP, Stallings VA, eds. *Finding a Path to Safety in Food Allergy: Assessment of the Global Burden, Causes, Prevention, Management, and Public Policy.* Washington (DC)2016.
38. Du Toit G, Roberts G, Sayre PH, et al. Randomized trial of peanut consumption in infants at risk for peanut allergy. *N Engl J Med.* 2015;372(9):803-813.
39. Togias A, Cooper SF, Acebal ML, et al. Addendum guidelines for the prevention of peanut allergy in the United States: Report of the National Institute of Allergy and Infectious Diseases-sponsored expert panel. *J Allergy Clin Immunol.* 2017;139(1):29-44.
40. Pistiner M, Mendez-Reyes JE, Eftekhari S, et al. Caregiver-Reported Presentation of Severe Food-Induced Allergic Reactions in Infants and Toddlers. *J Allergy Clin Immunol Pract.* 2021;9(1):311-320 e312.
41. Simons FE, Sampson HA. Anaphylaxis: Unique aspects of clinical diagnosis and management in infants (birth to age 2 years). *J Allergy Clin Immunol.* 2015;135(5):1125-1131.
42. Rudders SA, Banerji A, Clark S, Camargo CA, Jr. Age-related differences in the clinical presentation of food-induced anaphylaxis. *J Pediatr.* 2011;158(2):326-328.
43. Mikhail I, Stukus DR, Prince BT. Fatal Anaphylaxis: Epidemiology and Risk Factors. *Curr Allergy Asthma Rep.* 2021;21(4):28.
44. Bilo MB, Martini M, Tontini C, Corsi A, Antonicelli L. Anaphylaxis. *Eur Ann Allergy Clin Immunol.* 2021;53(1):4-17.
45. Toy D, Braga MS, Greenhawt M, Shaker M. An update on allergic emergencies. *Curr Opin Pediatr.* 2019;31(3):426-432.
46. Jansen van Rensburg S, Franken A, Du Plessis JL. Measurement of transepidermal water loss, stratum corneum hydration and skin surface pH in occupational settings: A review. *Skin Res Technol.* 2019;25(5):595-605.
47. Hendricks AJ, Eichenfield LF, Shi VY. The impact of airborne pollution on atopic dermatitis: a literature review. *Br J Dermatol.* 2019.
48. Alexander H, Brown S, Danby S, Flohr C. Research Techniques Made Simple: Transepidermal Water Loss Measurement as a Research Tool. *J Invest Dermatol.* 2018;138(11):2295-2300 e2291.

49. Wilson D, Berardesca E, Maibach HI. In vitro transepidermal water loss: differences between black and white human skin. *Br J Dermatol.* 1988;119(5):647-652.
50. Grimes P, Edison BL, Green BA, Wildnauer RH. Evaluation of inherent differences between African American and white skin surface properties using subjective and objective measures. *Cutis.* 2004;73(6):392-396.
51. Voegeli R, Rawlings AV, Summers B. Facial skin pigmentation is not related to stratum corneum cohesion, basal transepidermal water loss, barrier integrity and barrier repair. *Int J Cosmet Sci.* 2015;37(2):241-252.
52. Wesley NO, Maibach HI. Racial (ethnic) differences in skin properties: the objective data. *Am J Clin Dermatol.* 2003;4(12):843-860.
53. Peer RP, Burli A, Maibach HI. Did human evolution in skin of color enhance the TEWL barrier? *Arch Dermatol Res.* 2021.
54. Berardesca E, Loden M, Serup J, Masson P, Rodrigues LM. The revised EEMCO guidance for the in vivo measurement of water in the skin. *Skin Res Technol.* 2018;24(3):351-358.
55. Leung DYM, Calatroni A, Zaramela LS, et al. The nonlesional skin surface distinguishes atopic dermatitis with food allergy as a unique endotype. *Sci Transl Med.* 2019;11(480).
56. Ashley SE, Tan HT, Vuillermin P, et al. The skin barrier function gene SPINK5 is associated with challenge-proven IgE-mediated food allergy in infants. *Allergy.* 2017;72(9):1356-1364.
57. Kelleher M, Culliane C, Dunn Galvin A, Murray D, Hourihane JOB, Team B. Early life Transepidermal Water Loss (TEWL) values as a predictor of food allergy and sensitisation at 2 years: results from the BASELINE Study. *Clinical and Translational Allergy.* 2015;5(3):O2.
58. Triggiani M, Patella V, Staiano RI, Granata F, Marone G. Allergy and the cardiovascular system. *Clin Exp Immunol.* 2008;153 Suppl 1:7-11.
59. Luo J, Chen Q, Min S, Yu J. Perioperative Anaphylaxis from a Perspective of Temperature. *J Invest Surg.* 2021:1-9.
60. Makabe-Kobayashi Y, Hori Y, Adachi T, et al. The control effect of histamine on body temperature and respiratory function in IgE-dependent systemic anaphylaxis. *J Allergy Clin Immunol.* 2002;110(2):298-303.
61. Manzano-Szalai K, Pali-Scholl I, Krishnamurthy D, Stremnitzer C, Flaschberger I, Jensen-Jarolim E. Anaphylaxis Imaging: Non-Invasive Measurement of Surface Body Temperature and Physical Activity in Small Animals. *PLoS One.* 2016;11(3):e0150819.
62. Kind LS. Fall in rectal temperature as an indication of anaphylactic shock in the mouse. *J Immunol.* 1955;74(5):387-390.
63. Greenhawt M, Shaker M, Wang J, et al. Peanut allergy diagnosis: A 2020 practice parameter update, systematic review, and GRADE analysis. *J Allergy Clin Immunol.* 2020;146(6):1302-1334.
64. Bernstein IL, Li JT, Bernstein DI, et al. Allergy diagnostic testing: an updated practice parameter. *Ann Allergy Asthma Immunol.* 2008;100(3 Suppl 3):S1-148.
65. Dosanjh A. Infant anaphylaxis: the importance of early recognition. *J Asthma Allergy.* 2013;6:103-107.

66. Uppala R, Phungoen P, Mairiang D, Chaiyarit J, Techasatian L. Pediatric Anaphylaxis: Etiology and Predictive Factors in an Emergency Setting. *Glob Pediatr Health.* 2021;8:2333794X211011301.
67. Blazowski L, Majak P, Kurzawa R, Kuna P, Jerzynska J. A severity grading system of food-induced acute allergic reactions to avoid the delay of epinephrine administration. *Ann Allergy Asthma Immunol.* 2021.
68. LaHood NA, Patil SU. Food Allergy Testing. *Clin Lab Med.* 2019;39(4):625-642.
69. Ballmer-Weber BK, Beyer K. Food challenges. *J Allergy Clin Immunol.* 2018;141(1):69-71 e62.
70. Sampson HA, Gerth van Wijk R, Bindslev-Jensen C, et al. Standardizing double-blind, placebo-controlled oral food challenges: American Academy of Allergy, Asthma & Immunology-European Academy of Allergy and Clinical Immunology PRACTALL consensus report. *J Allergy Clin Immunol.* 2012;130(6):1260-1274.
71. Burks AW, Jones SM, Wood RA, et al. Oral immunotherapy for treatment of egg allergy in children. *N Engl J Med.* 2012;367(3):233-243.
72. Shaker MS, Wallace DV, Golden DBK, et al. Anaphylaxis-a 2020 practice parameter update, systematic review, and Grading of Recommendations, Assessment, Development and Evaluation (GRADE) analysis. *J Allergy Clin Immunol.* 2020;145(4):1082-1123.
