# Supplementary material for: Transepidermal Water Loss in Oral Food Challenges in Children With Peanut Allergy: A Randomized Clinical Trial
Source: JAMA Netw Open. 2025 Nov 14;8(11):e2543371. doi: 10.1001/jamanetworkopen.2025.43371 (PMC12619095; doi:10.1001/jamanetworkopen.2025.43371)
Supplement: Supplement 2. — eMethods. eFigure 1. Schematic of Study Design eFigure 2. Anaphylaxis Rates and Mean Scores in Nonsignificant Scoring Systems eFigure 3. Anaphylaxis Rates and Epinephrine Use in All Participants (Both Reactors and Nonreactors) by Intervention Group eFigure 4. Representative TEWL Tracings in Select Model Participants eTable. Summary of Baseline Participant Characteristics by Study Arm [file jamanetwopen-e2543371-s002.pdf]

Supplementary Online Content

Freigeh GE, O'Shea KM, Troost JP, Kaul B, Franco LM, Schuler CF IV. Transepidermal water loss in oral food challenges in children with peanut allergy: a randomized clinical trial. *JAMA Netw Open*. 2025;8(11):e2543371.
doi:10.1001/jamanetworkopen.2025.43371

eMethods

eFigure 1. Schematic of Study Design

eFigure 2. Anaphylaxis Rates and Mean Scores in Nonsignificant Scoring Systems

eFigure 3. Anaphylaxis Rates and Epinephrine Use in All Participants (Both Reactors and Nonreactors) by Intervention Group

eFigure 4. Representative TEWL Tracings in Select Model Participants

eTable. Summary of Baseline Participant Characteristics by Study Arm

This supplementary material has been provided by the authors to give readers additional information about their work.

© 2025 Freigeh GE et al. *JAMA Network Open*.

eMethods

Exclusion Criteria

Exclusion criteria included any cardiovascular disease, cancer, pulmonary disease except well-controlled asthma, or any other condition that would preclude an OFC. Individuals using a biologic medication or who used a biologic medication within the preceding three months were excluded. Participants undergoing oral immunotherapy for any foods were excluded.

Oral Food Challenge (OFC) Protocol

Participants were well on the day of the OFC without signs or symptoms of infection, did not have active asthma or AD exacerbation, and did not have a FA reaction within the previous four weeks. Participants were free from any medication use that would interfere with OFC outcome, most commonly oral antihistamine use within the previous seven days. Participants ingested escalating food doses of peanut butter every 15-20 minutes with a total of six doses ($1/16^{th}$ tsp, $1/8^{th}$ tsp, 1/4 tsp, 1/2 tsp, 1 tsp, 2 tsp).

Transepidermal Water Loss (TEWL) Measurement

We used a Tewameter VT310 device (Courage + Khazaka, Germany); these probes and supporting software were supplied at no cost and with no obligation except mention of material support in publications by the manufacturer. Data was collected in MPA CT Plus software (Courage + Khazaka, Germany) which provides note-taking functions and exports data into Microsoft Excel (Seattle, WA) for analysis.

Additional Statistics

To achieve 80% power with two-sided alpha=0.05 to detect a minimum 50% absolute difference in anaphylaxis rate reduction in anaphylaxis between the intervention group (anticipated anaphylaxis rate 30%) and the control group (80%) using a Fischer's exact test at a ratio of 1:1 for both groups, 38 total subjects (19 per group) needed to be recruited. To account for 5% drop-out, anticipated enrollment of 40 total subjects (20 per group).

For outcomes with statistically significant differences, a "worst-case scenario" analysis was conducted by imputing values for the four participants who dopped out before completing the food challenge. The intervention participants were imputed as having the worst level of the outcome; the two control participants were imputed as having the best level of the outcome.

© 2025 Freigeh GE et al. *JAMA Network Open*.

eFigure 1. Schematic of study design

The study population included participants aged 6 months to 5 years with a history of clinical peanut reaction and sensitization to peanut based on skin prick and/or serum IgE testing. Participants were then randomized to either the control or intervention group. All participants underwent peanut OFC using clinic protocols. The control group used stopping criteria based on traditional research-based OFC dose limiting symptoms. The intervention group used stopping criteria of either a single objective symptom plus a rise in 1 unit of TEWL or traditional research-based OFC dose limiting symptoms, whichever came first. All participants had TEWL data collected and adjudication of objective symptoms and dose limiting symptoms was done by a blinded allergist who did not have access to TEWL results. In the intervention group, an unblinded research coordinator indicated if a qualifying TEWL rise occurred and would stop the OFC if the blinded allergist independently identified an objective reaction symptom. The primary outcome measure was anaphylaxis rate in each group.

© 2025 Freigeh GE et al. *JAMA Network Open*.

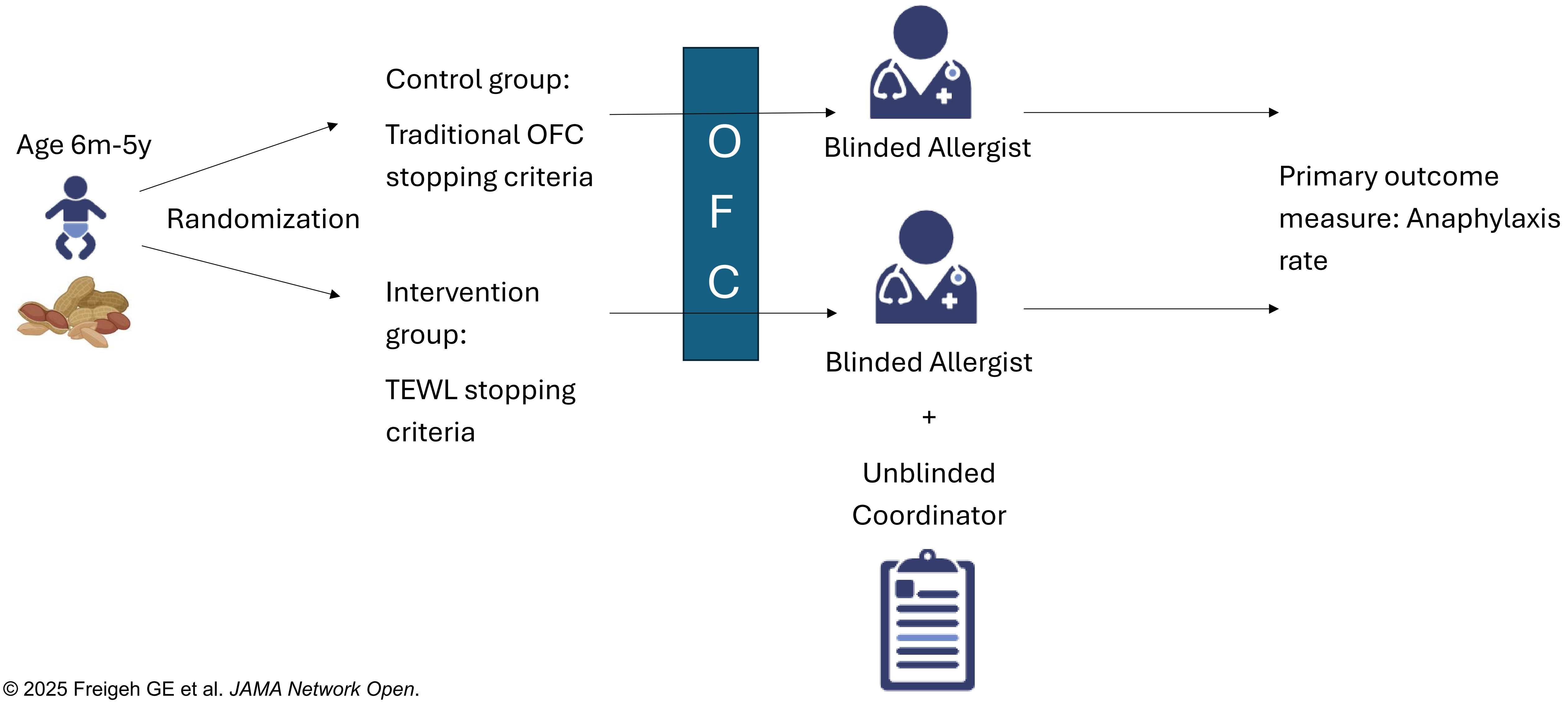

© 2025 Freigeh GE et al. *JAMA Network Open*.

eFigure 2. Anaphylaxis rates and mean scores in non-significant scoring systems

A shows a composite comparison of anaphylaxis rates by scoring system. B and C show anaphylaxis rates in reactors by Brighton score and distribution of Brighton scores. D and E show anaphylaxis rates in reactors by FAAN score and distribution of FAAN scores. Statistical analysis done with Fischer's exact test and unpaired *t* test.

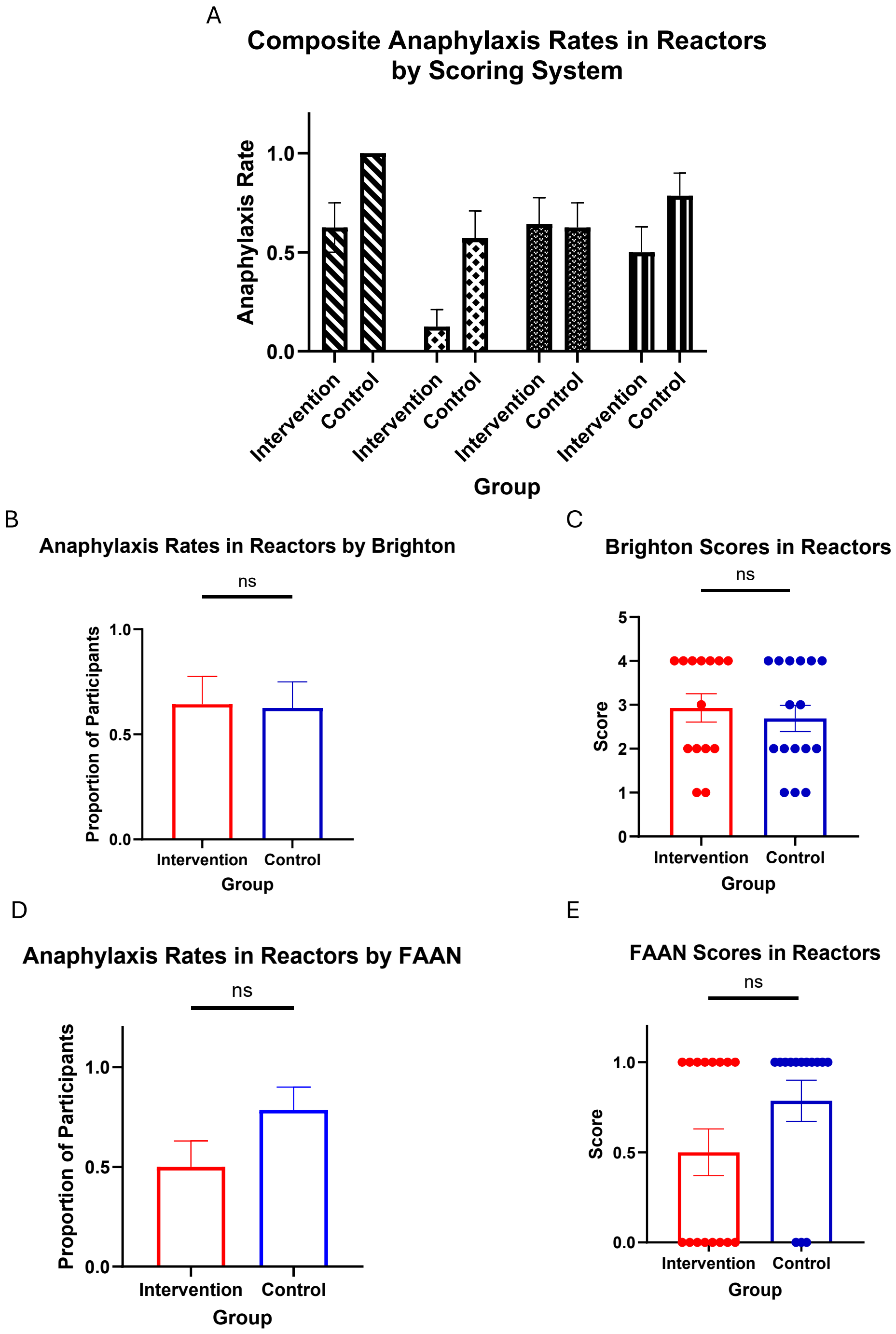

© 2025 Freigeh GE et al. *JAMA Network Open*.

eFigure 3. Anaphylaxis rates and epinephrine use in all participants (both reactors and non reactors) by intervention group. Statistical analysis done with Fischer's exact test.

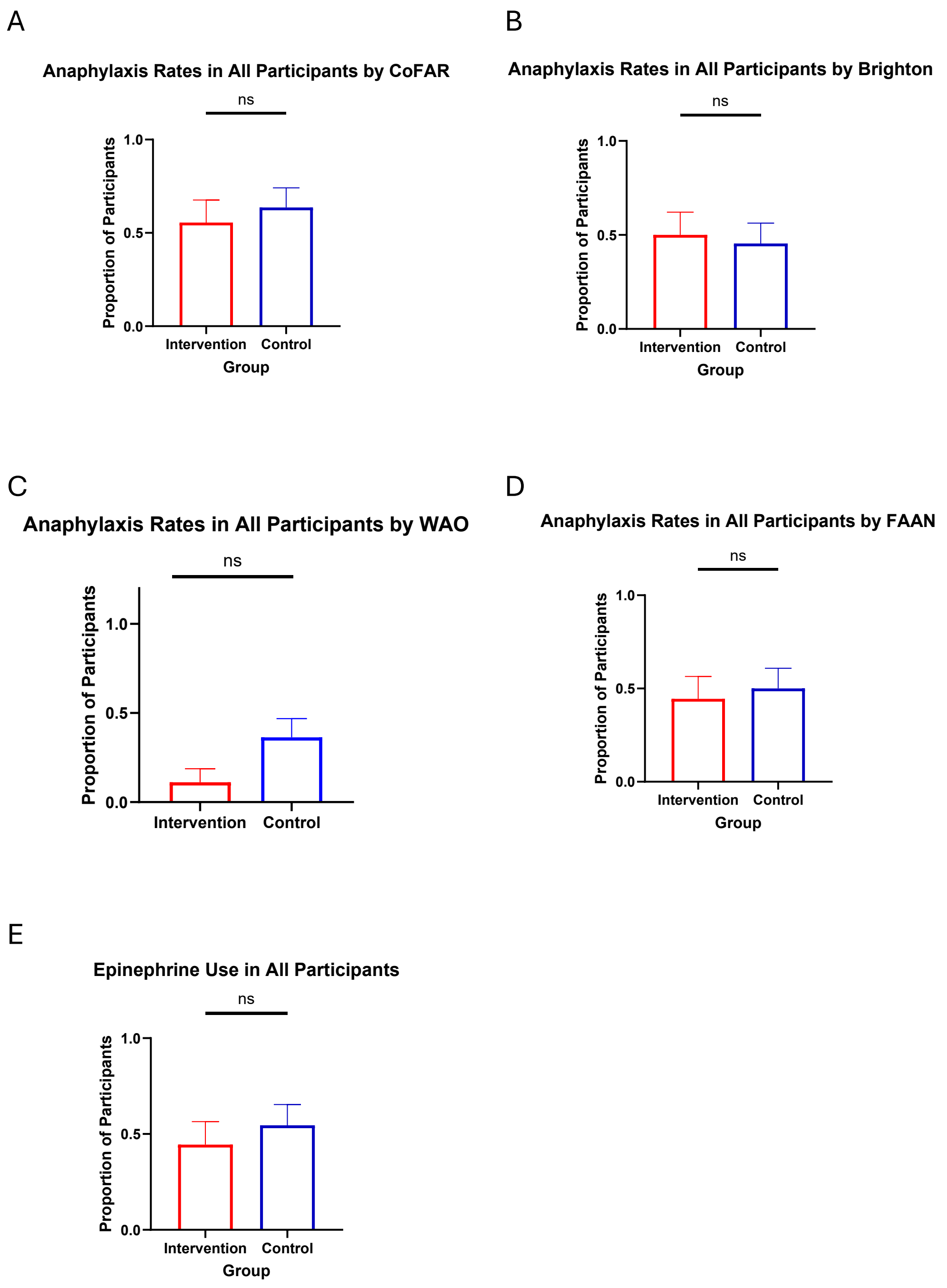

© 2025 Freigeh GE et al. *JAMA Network Open*.

eFigure 4. Representative TEWL tracings in select model participants

A shows a nonreactor, who does not have any demonstrable or consistent TEWL rise and also did not have any symptoms of food reaction. B shows a reactor in the intervention group with a TEWL rise at approximately minute 20 (dose 2), followed by an objective symptom at approximately minute 22. OFC was subsequently stopped. The participant was treated with an oral antihistamine and did not require epinephrine. C shows a reactor in the control group who did have TEWL rise. TEWL rise occurred immediately after the first dose was given and they had appearance of an objective symptom that was not dose limiting at approximately minute 21 (dose 2). They did not develop dose limiting symptom until approximately minute 44 (dose 3) and at that time OFC was stopped. They received oral antihistamine as well as injectable epinephrine. If this participant was in the intervention group, OFC would have been stopped after dose 2 given previous TEWL rise and objective symptom. D shows a reactor with a later TEWL rise at approximately minute 5 (not within 2 minutes of dose 1). They developed an objective symptom at minute 42 (dose 2) but since they did not have a qualifying TEWL rise the challenge was continued until dose 3 when dose limiting symptom occurred.

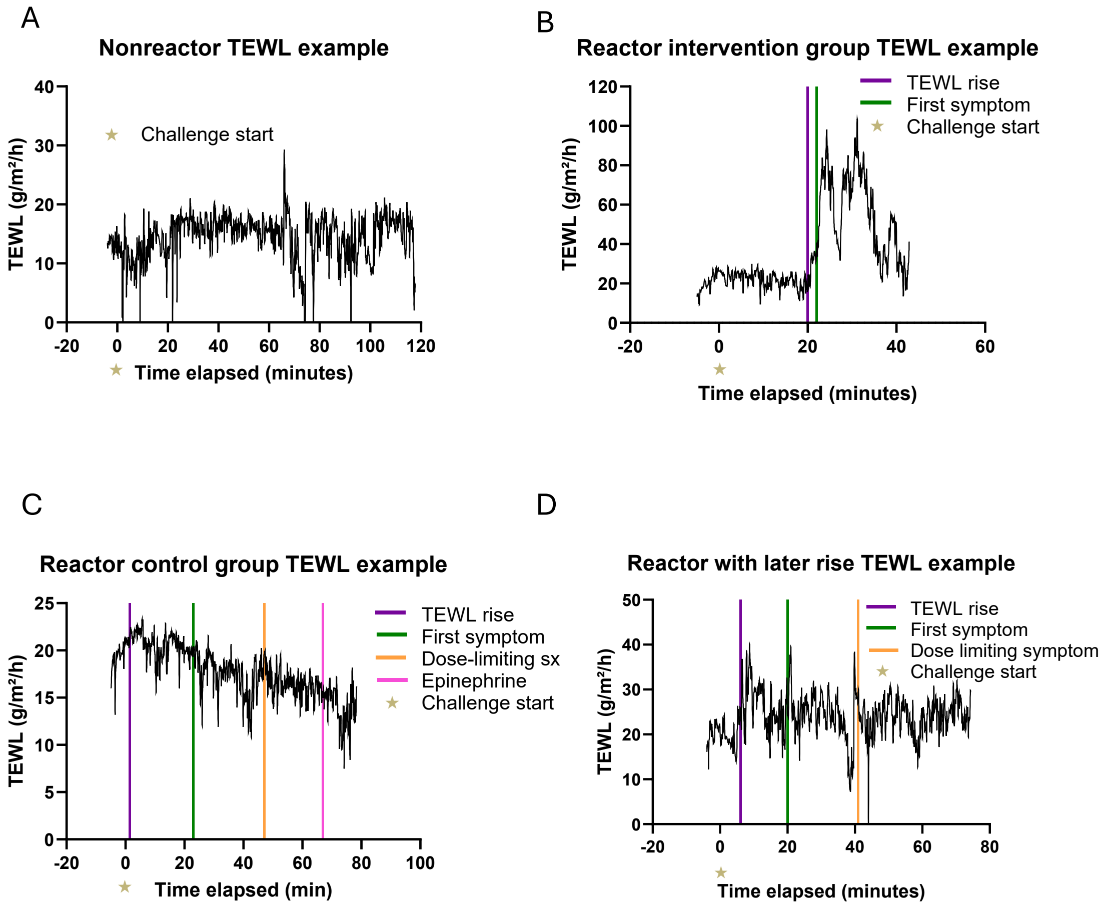

© 2025 Freigeh GE et al. *JAMA Network Open*.

Characteristic	Overall (No. %)	Intervention (No. %)	Control (No. %)
Age (in months), mean (SD)	31.8 (16.18)	34.6 (17.36)	29.5 (15.16)
Female sex	17 (43)	7 (39)	10 (45)
Race			
Asian	1 (3)	1 (6)	0 (0)
Black or African American	1 (3)	1 (6)	0 (0)
White	37 (93)	16 (89)	21 (95)
Other	1 (3)	0 (0)	1 (5)
Hispanic Ethnicity	2 (5)	0 (0)	2 (9)
Total peanut IgE, median (IQR)	1.3 (0.6 to 5.8)	1.3 (0.6 to 13.8)	1.4 (0.4 to 3.7)
nAra h 1, median (IQR)	0.1 (0.1 to 0.5)	0.1 (0.1 to 0.5)	0.1 (0.1 to 0.9)
nAra h 2, median (IQR)	0.8 (0.3 to 5.9)	1.4 (0.5 to 9.4)	0.6 (0.2 to 3.0)
nAra h 3, median (IQR)	0.1 (0.1 to 0.3)	0.1 (0.1 to 0.3)	0.1 (0.1 to 0.2)
nAra h 8, median (IQR)	0.1 (0.1 to 0.1)	0.1 (0.1 to 0.1)	0.1 (0.1 to 0.1)
Wheal, mean (SD)	7.2 (3.81)	5.1 (2.45)	8.8 (3.96)
Flare, mean (SD)	22.2 (10.06)	16.7 (10.41)	26.6 (7.37)

eTable. Summary of baseline participant characteristics by study arm. Race and ethnicity were determined by participant self-identification. The category of "other" included those that indicated more than one race.

© 2025 Freigeh GE et al. *JAMA Network Open*.
