# Supplementary material for: Transepidermal Water Loss in Oral Food Challenges in Children With Peanut Allergy: A Randomized Clinical Trial
Source: JAMA Netw Open. 2025 Nov 14;8(11):e2543371. doi: 10.1001/jamanetworkopen.2025.43371 (PMC12619095; doi:10.1001/jamanetworkopen.2025.43371)
Supplement: Supplement 3. — Data Sharing Statement [file jamanetwopen-e2543371-s003.pdf]

Data Sharing Statement

Freigeh. Transepidermal Water Loss in Oral Food Challenges in Children With Peanut Allergy. *JAMA Netw Open*. Published November 14, 2025. doi:10.1001/jamanetworkopen.2025.43371

Data
Additional Information: NCT05696236 Predicting Peanut Anaphylaxis and Reducing Epinephrine (PrePARE) https://clinicaltrials.gov/study/NCT05696236?term=schuler%20tewl&rank=1
Data available: Yes
Data types: Deidentified participant data
How to access data: All U-M data requests go through the Data Office. They can be contacted at DataOffice@umich.edu
When available: With publication

Supporting Documents
Document types: None

Additional Information
Who can access the data: Anyone requesting who can meet U-M's data office requirements consistent with the informed consent.
Types of analyses: Only for academic purposes.
Mechanisms of data availability: With investigator support.
